# Untargeted metabolomics reveals changes in boar sperm and seminal plasma metabolites associated with sexual maturity

**DOI:** 10.1186/s40104-025-01258-x

**Published:** 2025-09-03

**Authors:** Asmita Shrestha, Ann Helen Gaustad, Janne Beate Øiaas, Anna Nordborg, Elisabeth Kommisrud, Maren van Son, Terkel Hansen, Anne Hege Alm-Kristiansen

**Affiliations:** 1 Department of Biotechnology, CRESCO, Centre for Embryology and Healthy Development, University of Inland Norway, Holsetgata 31, 2318 Hamar, Norway; 2 Norsvin SA, Storhamargata 44, 2317 Hamar, Norway; 3 Department of Biotechnology and Nanomedicine, SINTEF Industry, Richard Birkelands Vei 3, Trondheim, Norway

**Keywords:** Global metabolomics, Seminal plasma, Sexual maturity, Spermatozoa, *Sus scrofa domestica*

## Abstract

**Background:**

Boars undergo physiological and biochemical changes in semen composition as they grow from puberty to sexual maturity. However, comprehensive metabolomic profiles of boar semen remain uncharacterised. Understanding metabolic alterations in semen during this period is important for optimising reproductive performance in breeding programs. The aim of this study was to characterise the semen metabolome as boars mature, utilising an untargeted metabolomic approach. Semen samples were collected from 15 Duroc boars at three developmental ages: ~ 7 months, 8.5 months, and 10 months. Sperm and seminal plasma were separated and analysed by hydrophilic interaction and reversed-phase liquid chromatography coupled with mass spectrometry to capture a wide range of metabolites.

**Results:**

We identified a total of 4,491 features in boar semen, annotating 92 distinct metabolites. Amino acids, peptides and analogues constituted the most abundant components, followed by fatty acid esters. Principal component analysis (PCA) and partial least squares discriminant analysis (PLS-DA) showed a clear separation between metabolomic profiles by age groups. PERMANOVA analysis of PCA scores confirmed statistically significant differences (*P* < 0.05) between younger (7 months) and more mature boars (8.5 months and 10 months). Pathway analysis identified porphyrin metabolism, taurine and hypotaurine metabolism, and glycerolipid metabolism as significantly enriched pathways in sperm, while glutathione and nitrogen metabolism were prominently enriched in seminal plasma. Using linear modelling, partial Spearman correlation and random forest analyses, we identified homoisovanillic acid as a key metabolite discriminating age groups in both sperm and seminal plasma. Additionally, L-glutamic acid, decanoyl-L-carnitine and N-(1,3-Thiazol-2-yl)benzenesulfonamide emerged as important sperm metabolites, while glyceric acid, myo-inositol, glycerophosphocholine, and several other compounds were identified as critical seminal plasma metabolites.

**Conclusion:**

This study provides a detailed characterisation of metabolic changes in Duroc boar semen during the transition from puberty to sexual maturity. Our findings enhance the understanding of reproductive development and could inform strategies to assess sexual maturity in breeding programs.

**Supplementary Information:**

The online version contains supplementary material available at 10.1186/s40104-025-01258-x.

## Introduction

Sexual maturity influences reproductive performance, genetic improvement, and overall productivity in swine production. Boars reach sexual maturity between three and seven months, with substantial breed variation influenced by genetic and environmental factors [1–3]. Early maturing breeds like Chinese Meishan and Portuguese Bisaro boars attain spermatogenesis by 2.5–3 months of age [4], with sexual capacity evident between 3.5 and 4.4 months of age [1, 3]. Modern commercial breeds do not reach full sexual maturity until approximately 7 months of age, and their fertility continues to increase substantially with age, reaching its peak between 9 and 20 months of age [4]. Typically, Duroc boars are reported to reach puberty at about 5 months [5], and sexual maturity by 9 months [3], with the highest sperm production at around 18 months [6, 7]. Early maturation enables the use of younger males for breeding, thereby shortening generation intervals and accelerating genetic progress [8].

As boars mature sexually, there is a marked increase in ejaculate volume and total sperm number per ejaculate, which are important for artificial insemination (AI) practices [9, 10]. It is reported that between 8 and 10 months of age, there is an increase in the sperm length, width and head area, along with a reduction in the percentage of sperm with minor abnormalities [11]. This maturation period also features increased testicular weight [12] and hormonal shifts [12, 13] driving the development of secondary sexual characteristics and libido [14].

Determining sexual maturity onset in boars presents several challenges. Traditional methods of assessment, such as physical examinations, hormonal assays, and assessment of semen quality, often have limitations. Physical examinations assess only external characteristics, which may not accurately reflect internal physiological readiness for reproduction. For example, Hezuo boars show libido at around 1.5 months, but develop abundant spermatogonia cells after 4 months [15]. Hormonal assays are influenced by various external factors such as stress and nutrition [16–19], leading to fluctuations in hormone levels, which may not reliably indicate sexual maturity. Parameters such as motility, morphology, and acrosome integrity are reported to vary significantly between boars and within ejaculates from the same boar [20], and their relationship to sexual maturity remains poorly characterised. There is no single parameter that defines the animals’ sexual maturity, as progression is gradual rather than an abrupt occurrence [21]. This highlights the need for more comprehensive approaches that could provide complementary information for effective sexual maturity assessment.

Although genetic companies are increasingly using younger males to accelerate genetic progress [22], selecting animals that have not yet achieved true reproductive maturity creates potential conflicts. Untargeted metabolomics is a powerful analytical method that offers thorough profiling of metabolites, which are end products of cellular processes in biological samples, without needing prior knowledge of which specific metabolites are present. This approach provides insights into physiological states, biochemical pathways and metabolic processes occurring within an organism [23, 24]. Zhao et al. utilised untargeted metabolomics to identify potential biomarkers in the small yellow follicle of chickens during sexual maturation, highlighting L-arginine, L-prolinamide, (R)-4-hydroxymandelate, glutathione and homovanillic acid as important metabolites in this process [25].

The sperm metabolite profile can provide insights into their functional capacity, including fertility [26, 27], motility and viability [28]. Studies have identified specific metabolites in sperm associated with fertility outcomes; for example, gamma-aminobutyric acid (GABA), carbamate, benzoic acid, lactic acid, and palmitic acid were reported to be significantly different between high-fertility and low-fertility bulls [26]. Similarly, cysteine and glutamic acid in sperm were reported to have a negative association with fertility, while aspartic acid, leucine and serine were reported to have a positive association with fertility in bulls [27]. Seminal plasma contains a rich array of metabolites that provide nutrients and protective factors to enhance sperm survival and help them navigate through the female reproductive tract [29]. Velho et al. reported an association of 2-oxoglutaric acid and fructose in seminal plasma with the fertility of bulls [30]. A study in humans comparing seminal plasma metabolites in young men aged 26 to 30 and older men aged 45 to 54 identified that age affects citrate cycle, oxidative phosphorylation, and glutathione metabolism [31]. This shows valuable insights into how age impacts seminal plasma composition and consequently male fertility.

Our recent targeted metabolomic analysis of amino acids and amines in boar semen identified key sexual maturity-related changes, particularly in glutamate, alanine, aspartate and choline [32]. However, this targeted approach captured only a fraction of the metabolic landscape, leaving the broader range of lipids, energy metabolites, antioxidants, and signalling molecules unexplored, highlighting the necessity of untargeted metabolomics research in this area.

The present study aimed to provide a comprehensive metabolomic characterisation of boar semen during sexual maturation, identifying key metabolites and pathways that change during this critical reproductive transition. This was performed through a longitudinal study design collecting semen samples from Duroc boars across three maturation time points (7, 8.5, and 10 months), and analysing sperm and seminal plasma separately by untargeted metabolomic analysis (LC–MS/MS platform). This holistic discovery profiling approach enabled the identification of metabolic fingerprints of reproductive development and major metabolic pathways involved in sexual maturation. Our work represents the first in-depth metabolomic exploration of boar sexual maturation, establishing a foundational knowledge base for future research and breeding strategies.

## Methods

### Study design and sample collection

Fifteen male Duroc swine (*Sus scrofa domesticus*) selected randomly from the commercial breeding line of Norsvin SA (Norway) were followed longitudinally over three months. Semen was collected repeatedly from these boars from March to June 2023, encompassing three growth phases: 7.16 ± 0.44 months (designated as 7 months), 8.56 ± 0.46 months (designated as 8.5 months), and 9.94 ± 0.47 months (designated as 10 months).

During the first semen collection at 7 months of age, the boars were kept in individual pens measuring 4 m^2^ within an isolation unit, as approved by the Norwegian Food Safety Authority, where they were trained for semen collection. Semen collection began as soon as the boars showed interest in mounting the dummy and were physically able to produce an ejaculate. The same boars were transferred to an Artificial Insemination (AI) centre at 8.5 months of age, where they were housed in larger pens of 6 m^2^. In compliance with Council Directive 90/429/EEC, Norsvin SA commences commercial semen distribution from boars from approximately 8.5 months of age.

Throughout both the isolation unit and the AI centre, consistent housing conditions, including ventilation, lighting and temperature, were maintained. All the boars received the nutritionally balanced commercial diet. The semen collection was performed using the gloved-hand technique by the same trained personnel for all boars to maintain consistency. The boars were briefly moved from their home pens to a separate area for collection. This longitudinal design with repeated sampling from the same individuals allowed us to capture true developmental changes while minimising random inter-individual variability.

### Semen samples

Sperm concentration of the collected samples was assessed at the isolation unit and AI station using NucleoCounter^®^ SP-100™ (Chemometec, Allerød, Denmark). Semen samples were diluted in Androstar^®^ Plus extender (Minitube, Germany) to 25 million sperm/ml and incubated at 38 °C for 20 min before assessment of motility by Sperm Class Analyzer (Microptic SL, Spain). Spermatozoa were identified by head area 20–80 μm^2^ and the defined threshold for motility was velocity average path > 10 µm/s. The samples were transported in Styrofoam boxes to the laboratory within 30 min. For spermatozoa isolation, 200 million sperm cells from each raw semen sample were centrifuged at 600 × *g* for 10 min at room temperature, and the supernatant was discarded. Seminal plasma was separated from the raw semen sample and prepared through a two-step centrifugation protocol (600 × *g* at room temperature and 10,000 × *g* at 4 °C) for 10 min. Thereafter, each seminal plasma sample was carefully examined under a microscope across multiple fields of view to confirm the complete absence of spermatozoa. Both separated sperm pellet and seminal plasma were snap-frozen in liquid nitrogen and stored at −80 °C until metabolomic analysis.

### Untargeted metabolomics profiling

Untargeted metabolomics profiling was conducted by high-performance liquid chromatography (Agilent 1290, Agilent, Santa Clara, CA, USA) coupled to a high-resolution quadrupole time-of-flight mass spectrometer (Bruker Impact II QTOF, Bruker, Bremen, Germany).

Sample extraction was conducted by an in-house modified SIMPLEX extraction [33] for the removal of lipophilic components and isolation of a hydrophilic phase for metabolome analysis. For the seminal plasma, a sample volume of 100 µL was utilised. For the spermatozoa samples, the isolated spermatozoa pellet was redissolved in 500 µL of PBS buffer prior to sample extraction, and a volume of 100 µL was utilised in the sample work-up. To isolate the metabolites, the lipophilic components were first removed by extraction with a combination of methanol (225 µL ice-cold methanol) and 750 µL methyl *tert*-butyl ether, added sequentially with the sample vortexed between additions. Finally, 400 µL of water was added, followed by another vortex for 20 s. After centrifugation at 14,000 × *g* for 2 min at 10 °C, the major portion of the 400 µL of the upper lipophilic phase was removed before the addition of 50 µL chloroform, followed by a 10-s vortex, causing an inversion of the lipophilic and hydrophilic phases. 100 µL of the upper hydrophilic phase was collected for analysis, evaporated to dryness, and resolubilised in 50 µL methanol prior to analysis.

Mass spectrometric data were obtained from a Bruker Impact II mass spectrometer coupled to an Agilent 1290 HPLC. All data was acquired utilising a data-dependent acquisition method with a cycle time of 0.5 s. Two chromatographic methods were utilised, one reverse-phase positive ion mode (RP-Pos) method using a Waters BEH C18 (2.1 mm × 50 mm) column and one separation in hydrophilic interaction liquid chromatography (HILIC) mode employing a Waters BEH amide (2.1 mm × 50 mm) column. Data for reversed-phase mode chromatographic separation of metabolites was acquired in positive ionisation mode, while negative ionisation mode was employed for the HILIC separation. Feature extraction, initial data quality control and metabolite annotation were conducted utilising Bruker MetaboScape software. Annotation is based on a match of observed mass and fragments against the NIST metabolite database integrated in the MetaboScape software.

Annotated metabolites were grouped into chemical classes (superclass) by ClassyFire [34].

### Statistical analysis

All statistical analyses of metabolites were performed using MetaboAnalyst 6.0 [35], MetaboAnalystR 4.0 [36] and R 4.4.2 [37]. Metabolite data were analysed separately for spermatozoa and seminal plasma to account for the distinct sample types.

For both the identified metabolites in spermatozoa and seminal plasma, data preprocessing before statistical analysis included normalisation, transformation, and scaling steps. The preprocessing steps were chosen based on a systematic evaluation using MetaboAnalyst 6.0, with different methods selected for each dataset to achieve optimal normalisation results. Normalisation effectiveness was evaluated using MetaboAnalyst’s integrated diagnostic tools, combining visual assessment of distribution density plots and variance box plots with quantitative metrics including skewness and variance stabilisation. For the spermatozoa RP-Pos data and HILIC dataset, all features were normalised by sum, log10 transformation, and autoscaling. Similarly, annotated spermatozoa metabolites from RP-Pos were normalised by log10 transformation and autoscaling, whereas those from HILIC were normalised by the sum, log10 transformation and mean-centering.

Likewise, all features in seminal plasma from RP-Pos were normalised by sum, log10 transformed, and autoscaled, whereas HILIC data were normalised by sum, log10 transformed, and Pareto-scaled. For annotated metabolites in the seminal plasma, data from both RP-Pos and HILIC were normalised to the sample median, log10 transformed, and autoscaled before statistical analysis. For all discovered features, principal component analysis (PCA) was performed to assess clustering patterns, and a pairwise PERMANOVA test was conducted on PCA scores to evaluate the statistical significance of group differences. The clustering pattern of metabolites was assessed using partial least squares discriminant analysis (PLS-DA) models. The model performance was evaluated using R^2^ and Q^2^ values obtained through cross-validation. Model validity was further confirmed using a permutation test (2000 permutations) based on the separation distance.

Annotated metabolites were analysed using the *limma* package in R [38]. Age groups were modelled as a fixed effect, and each boar’s three measurements were treated as a block via *limma*’s *duplicateCorrelation* to estimate within-animal correlation. Family relationships were included as a fixed covariate in the design matrix. ANOVA-style contrasts (8.5 months vs. 7 months, 10 months vs. 7 months, 10 months vs. 8.5 months) were tested for overall differences across time points. Additionally, partial Spearman rank correlations were computed on annotated metabolites using the *Correlation and Partial Correlation Analysis module.* The analysis assessed monotonic associations between metabolite abundance levels and longitudinal time points (age groups), independent of boars’ influence. The Benjamini–Hochberg method was applied to adjust for multiple testing, with adjusted *P*-values < 0.05 considered statistically significant. Random forest analysis was conducted with 5,000 trees and six predictors, and the model accuracy was evaluated using the out-of-bag (OOB) error rate. Annotated metabolites were ranked for their importance based on the mean decrease accuracy (MDA) value.

Sperm quality traits were handled separately in R 4.4.2 [37]. Concentration was fitted with a Gamma mixed-effects model and a log link, and motility with a binomial logit mixed-effects model, both run in *lme4* 1.1–36 [39]. Age was used as a fixed effect, individual boar and parents were used as random effects, capturing shared genetics and repeated measures. Pairwise age contrasts were extracted with *emmeans* 1.10.7 [40], and their *P*-values were adjusted with Benjamini–Hochberg method. Model fit and residual patterns were checked with *DHARMa* 0.4.7 [41] simulated residuals.

### Pathway analysis

Pathway analysis was performed on all identified features in MetaboAnalyst 6.0 [35] using Mummichog version 2 with a *P*-value cutoff of 0.05. The *Sus scrofa* KEGG database was used as a reference for the pathway mapping. Pathway enrichment and topology analyses were conducted to identify significantly altered metabolic pathways, with significance determined based on pathway impact scores and pathway *P*-values, aligning with the significance threshold set at an adjusted *P*-value < 0.05.

## Results

Sperm quality was slightly different between the age groups. Sperm concentration declined significantly from 7 months to 8.5 months and 10 months of age, while motility increased in the same time interval (Table 1, adjusted *P* < 0.05).

**Table 1:** Sperm concentration and percentage motility in boar spermatozoa

Parameter	Age	Mean ± SD	Median	Range
Concentration, 10^6^/mL	7 months	466.80 ± 194.50^a^	508.30	156.70–757.10
	8.5 months	316.90 ± 117.50^b^	311.00	125.00–511.90
	10 months	352.40 ± 138.10^b^	315.30	107.10–651.40
Motility, %	7 months	85.87 ± 9.38^a^	89.43	55.23–92.76
	8.5 months	91.97 ± 1.59^b^	91.98	89.87–95.31
	10 months	90.73 ± 3.22^b^	91.37	83.92–94.63

Semen samples were collected from 15 Duroc boars at three time points: initially when the animals were approximately 7 months old, followed by subsequent collections at 8.5 and 10 months. Values represent mean ± standard deviation, with median and range provided for each parameter. Within age, values sharing a letter are not significantly different (GLMM post hoc, adjusted *P* ≥ 0.05). Values with different letters differ significantly (GLMM post hoc, adjusted *P* < 0.05)

### Global metabolomic profiling

A total of 4,491 features were detected across all samples in both sample types, spermatozoa and seminal plasma. Specifically, 2,410 and 2,251 features were identified using RP-Pos and HILIC methods, respectively. Of these, 92 metabolites were successfully annotated using the NIST metabolite database integrated in the MetaboScape software, of which 65 were from RP-Pos and 27 were from HILIC.

**Fig. 1:**
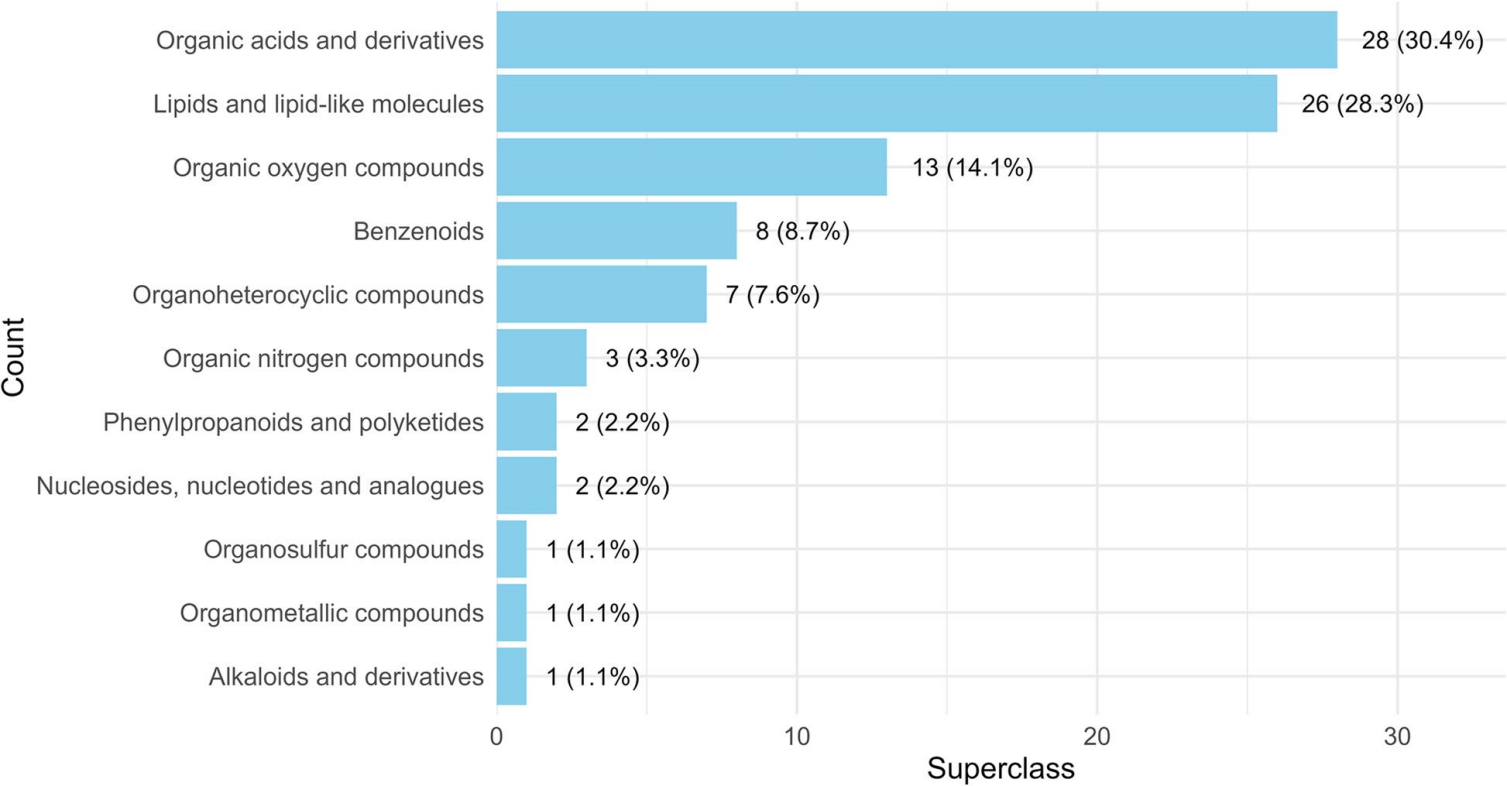
Chemical superclasses of annotated metabolites in boar semen with percentage distribution. Annotated metabolites were classified into chemical superclasses based on their structural properties. The bar plot displays the percentage and absolute number of metabolites identified in each superclass. Semen samples were collected from 15 Duroc boars, and spermatozoa were isolated from the seminal plasma for metabolomic analysis. Metabolite abundances were measured using liquid chromatography-mass spectrometry (LC–MS) with HILIC and RP-Pos chromatographic methods. Data from both HILIC and RP-Pos were merged for this analysis. The same annotated metabolites were observed in both spermatozoa and seminal plasma

The annotated metabolites were grouped into various chemical superclasses, including organic acids and derivatives, lipids and lipid-like molecules, organic oxygen compounds, benzenoids, organoheterocyclic compounds, organic nitrogen compounds, nucleosides, nucleotides and analogues, phenylpropanoids and polyketides, alkaloids and derivatives, organometallic compounds and organosulfur compounds (Fig. 1). Amino acids, peptides and analogues (*n* = 13) were the predominant compounds in organic acids and derivatives. Likewise, fatty acid esters (*n* = 10) were the most abundant, followed by glycerophosphocholines (*n* = 6) in the lipids and lipid-like molecules superclass. Detailed information can be found in Table S1 (Additional file 1).

### Multivariate assessment of semen metabolome

**Fig. 2:**
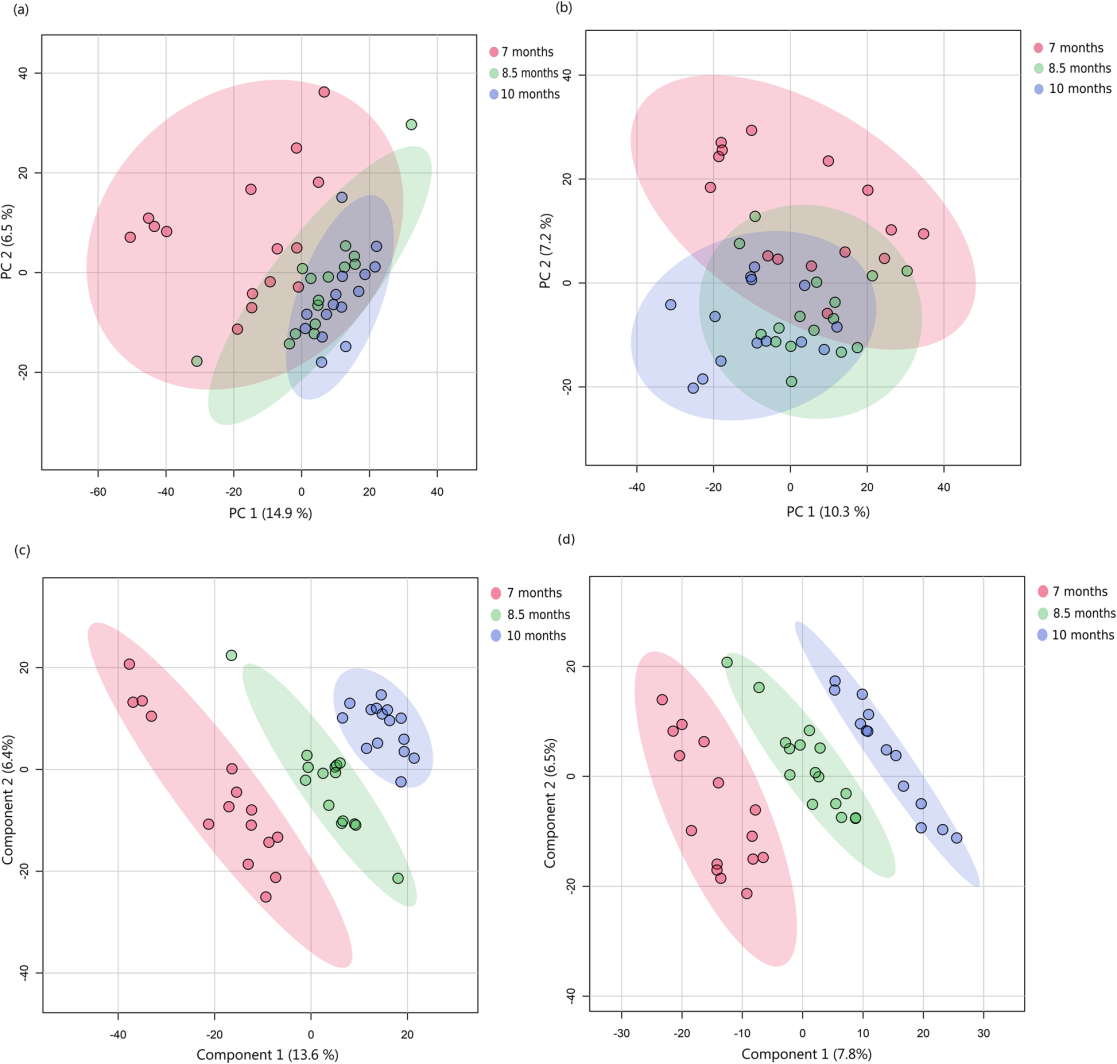
Score plots from global metabolomic profiling of boar sperm across three ages. Principal Component Analysis (PCA) score plots: (**a**) based on HILIC method and (**b**) based on RP-Pos method, showed metabolomic profiles with greater variability in 7-month-old boars, while Partial Least Squares Discriminant Analysis (PLS-DA) score plots: (**c**) based on HILIC method and (**d**) based on RP-Pos method showed effective classification of age groups. Semen samples were collected from 15 Duroc boars at three time points: initially when the animals were approximately 7 months old, followed by subsequent collections at 8.5 and 10 months. Spermatozoa were isolated from the seminal plasma, and metabolite abundances were measured using liquid chromatography-mass spectrometry (LC–MS) with HILIC and RP-Pos methods

**Fig. 3:**
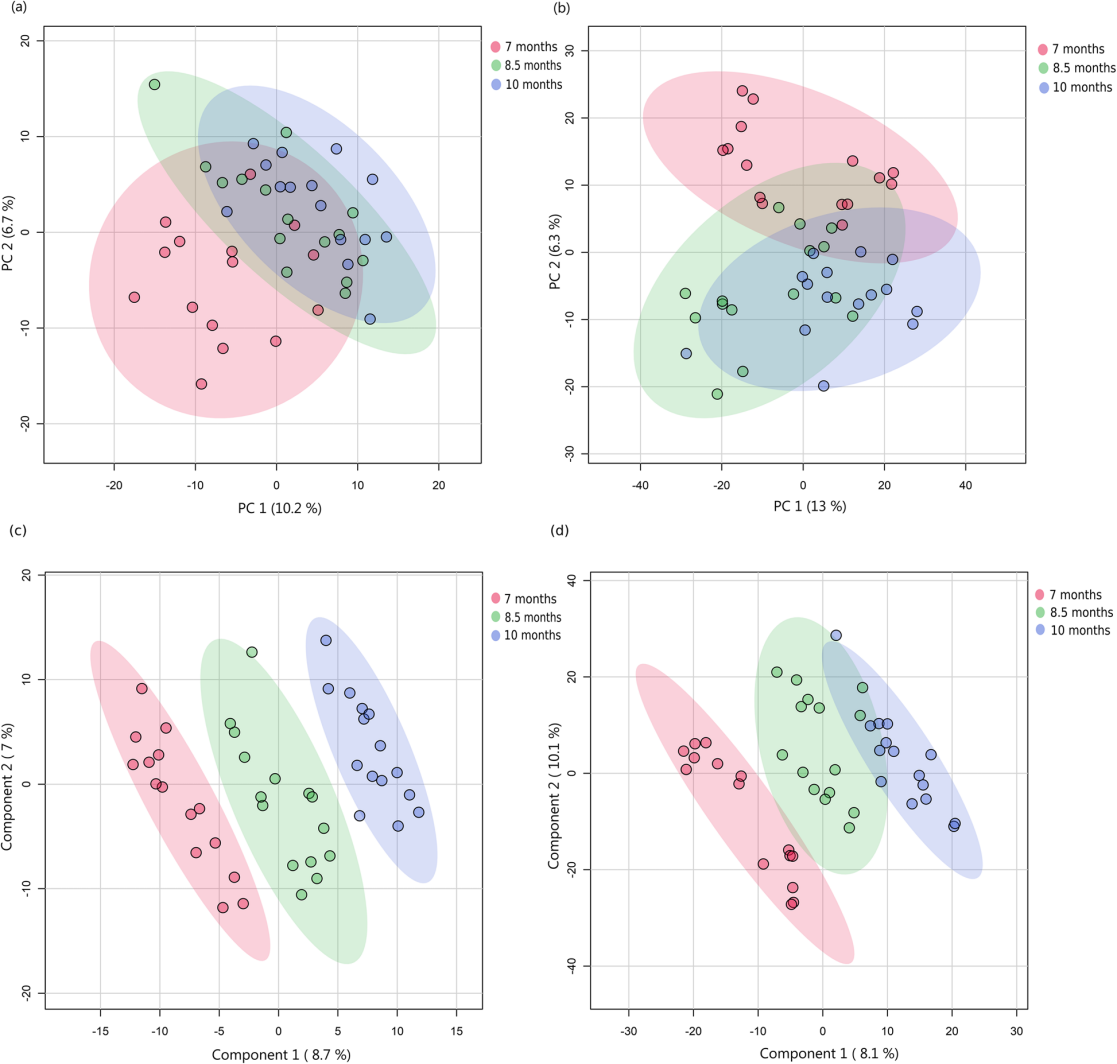
Score plots from global metabolomic profiling of boar seminal plasma across three ages. Principal Component Analysis (PCA) score plots: (**a**) based on HILIC method and (**b**) based on RP-Pos method, showed metabolomic profiles with greater variability in 7-month-old boars, while Partial Least Squares Discriminant Analysis (PLS-DA) score plots: (**c**) based on HILIC method and (**d**) based on RP-Pos method showed effective classification of age groups. Semen samples were collected from 15 Duroc boars at three time points: initially when the animals were approximately 7 months old, followed by subsequent collections at 8.5 and 10 months. Seminal plasma was separated from spermatozoa, and metabolite abundances were measured using liquid chromatography-mass spectrometry (LC–MS) with HILIC and RP-Pos methods

In both spermatozoa and seminal plasma, PCA score plots (Figs. 2a, b and 3a, b) showed intriguing metabolomic profile patterns across age groups in both the RP-Pos and HILIC datasets. The first two principal components (PC1 and PC2) explained a total of 21.3% and 17.5% of the variance in the HILIC and RP-Pos datasets for spermatozoa, respectively (Fig. 2a and b). Seminal plasma showed comparable variance explanation, with PC1 and PC2 accounting for 16.9% and 19.3% of the variance in the HILIC dataset and RP-Pos dataset, respectively (Fig. 3a and b).

Across both sample types, 7 months displayed higher metabolomic variability compared to those from 8.5 and 10 months. PERMANOVA analysis of the PCA scores revealed significant differences in metabolomic dispersion between younger boars (7 months) and both older age groups (adjusted *P* < 0.01) for all datasets examined. The greater variability in the youngest cohort was particularly pronounced, while samples from 8.5-month and 10-month-old boars clustered more tightly.

The PLS-DA model score plot (Fig. 2c and d) showed effective separation of the three ages in spermatozoa. The RP-Pos dataset showed strong predictive capability (accuracy = 0.76, R^2^ = 0.99, Q^2^ = 0.64), while the HILIC dataset showed even better classification capability (accuracy = 0.84, R^2^ = 0.99, Q^2^ = 0.80). Seminal plasma analysis yielded similar results. The score plots from PLS-DA (Fig. 3c and d) demonstrated a distinct separation between the three ages for both LC–MS methods, with model performance indicating a strong predictive capability for the HILIC dataset (accuracy = 0.87, R^2^ = 0.99, Q^2^ = 0.77). For the RP-Pos dataset, it showed good predictive ability with a Q^2^ of 0.62, higher classification accuracy (84%) and goodness of fit (R^2^ = 0.98).

While PCA revealed variability differences between age groups, supervised PLS-DA modelling could effectively distinguish age-related metabolomic patterns.

### Pathway analysis of metabolites in semen

Pathway analysis with all features of spermatozoa identified several pathways that differed among age groups, of which three were significantly enriched in sperm (*P* < 0.05). These included porphyrin metabolism (*P* = 0.002, enrichment factor = 6.25), taurine and hypotaurine metabolism (*P* = 0.006, enrichment factor = 12.5), and glycerolipid metabolism (*P* = 0.006, enrichment factor = 3.0). In seminal plasma, two pathways were significantly enriched (*P* < 0.05). These included glutathione metabolism (*P* = 0.03, enrichment factor = 2.57) and nitrogen metabolism (*P* = 0.03, enrichment factor = 2.57). Beyond these significant pathways, numerous additional pathways were also identified, showing age-related variations. The complete pathway analysis of spermatozoa and seminal plasma is detailed in Table S2 (Additional file 2) and Table S3 (Additional file 3), respectively.

### Identification of key metabolites

**Fig. 4:**
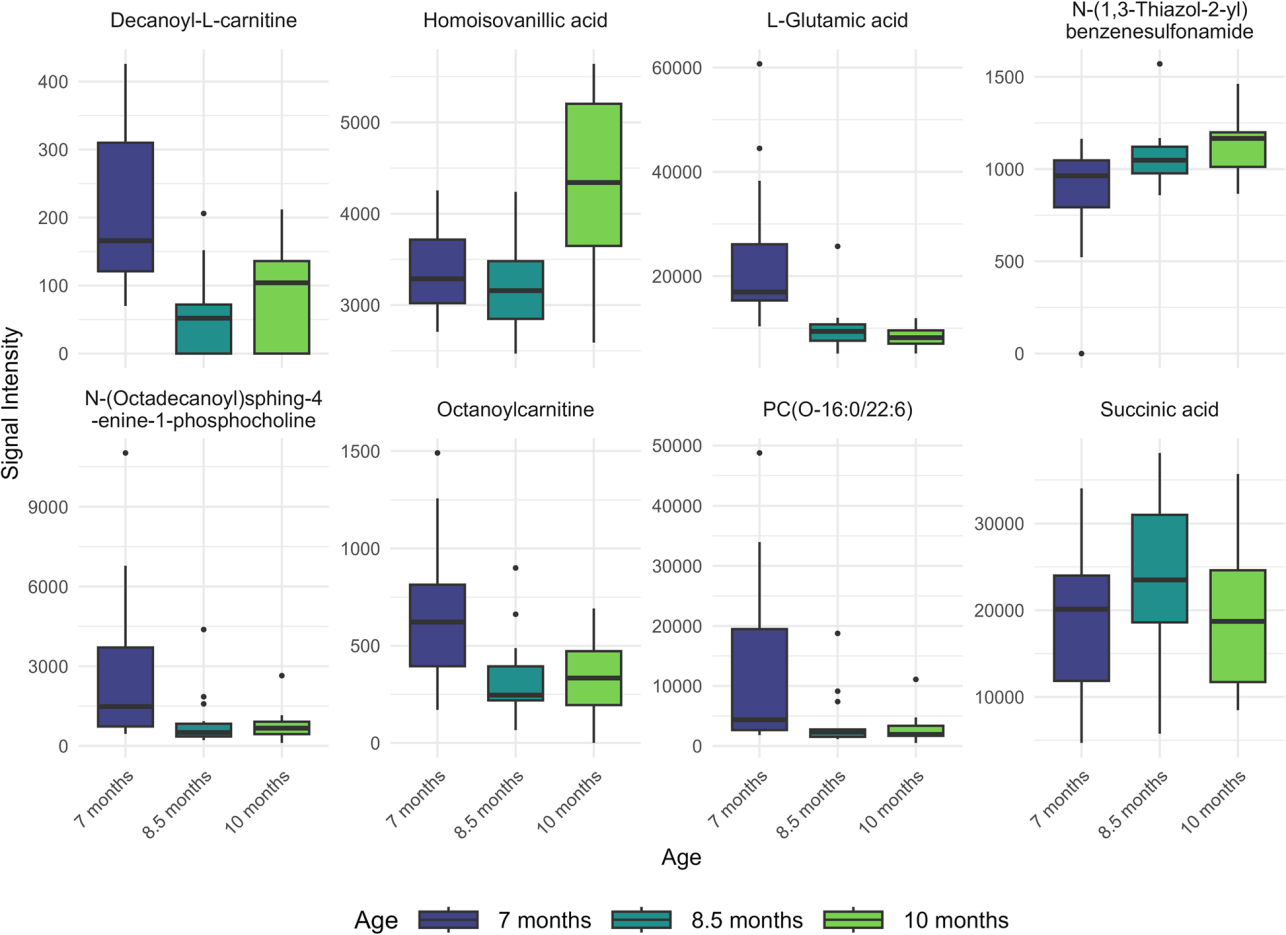
Longitudinal changes in boar sperm metabolite abundance during sexual maturation. Box plots display the distribution of eight significant metabolites across three ages in boar spermatozoa. The *x*-axis denotes age groups as 7 months, 8.5 months and 10 months, while the *y*-axis indicates the abundance of each metabolite. Semen samples were collected from 15 Duroc boars at three time points: initially when the animals were approximately 7 months old, followed by subsequent collections at 8.5 and 10 months. Spermatozoa were isolated from the seminal plasma, and metabolite abundances were measured using liquid chromatography-mass spectrometry (LC–MS) with HILIC and RP-Pos methods

In spermatozoa, the longitudinal analysis of metabolite intensity levels across boar sexual development revealed eight annotated metabolites with significant changes across ages (Fig. 4 and Table 2). L-Glutamic acid and decanoyl-L-carnitine showed the pronounced statistical significance, with consistent decreases at both 8.5 months and 10 months compared to 7 months (adjusted *P* < 0.05). Octanoylcarnitine also showed a decrease at 10 months (adjusted *P* < 0.05). Among the phospholipid species, both PC(O-16:0/22:6) and N-(octadecanoyl)sphing-4-enine-1-phosphocholine showed significant decreases at 10 months compared to 7 months (adjusted *P* < 0.05).

**Table 2:** List of annotated metabolites with significant abundance changes in boar spermatozoa across ages

Metabolite	Contrast	logFC	Fold change	SE	*t*-statistic	Adjusted *P* value
L-Glutamic acid	10 M vs. 7 M	−0.24	1.18	0.04	−5.75	4.11 × 10^–5^
L-Glutamic acid	8.5 M vs. 7 M	−0.25	1.19	0.04	−5.89	2.66 × 10^–5^
Decanoyl-L-carnitine	10 M vs. 7 M	−0.97	1.96	0.34	−2.86	3.33 × 10^–2^
Decanoyl-L-carnitine	8.5 M vs. 7 M	−1.31	2.48	0.34	−3.84	4.96 × 10^–3^
PC(O-16:0/22:6)	10 M vs. 7 M	−1.10	2.14	0.34	−3.22	2.84 × 10^–2^
Octanoylcarnitine	10 M vs. 7 M	−0.98	1.97	0.34	−2.89	3.33 × 10^–2^
N-(Octadecanoyl)sphing-4-enine-1-phosphocholine	10 M vs. 7 M	−1.02	2.03	0.34	−3.01	3.33 × 10^–2^
Succinic acid	8.5 M vs. 7 M	0.19	1.14	0.06	3.27	3.23 × 10^–2^
N-(1,3-Thiazol-2-yl)-benzenesulfonamide	10 M vs. 7 M	0.30	1.23	0.07	4.13	2.78 × 10^–3^
Homoisovanillic acid	10 M vs. 7 M	1.08	2.11	0.34	3.19	2.84 × 10^–2^
Homoisovanillic acid	10 M vs. 8.5 M	1.33	2.52	0.34	3.91	3.56 × 10^–3^

Fold changes are calculated as 2^logFC^. Ages correspond to 7 months (7 M), 8.5 months (8.5 M), and 10 months (10 M). SE stands for standard error. Semen samples were obtained from 15 Duroc boars at three time points: initially when the animals were approximately 7 months, followed by subsequent collections at 8.5 and 10 months. Spermatozoa were separated from seminal plasma, and liquid chromatography-mass spectrometry (LC–MS) with HILIC and RP-Pos methods was used to measure metabolite abundances in spermatozoa. Metabolite differences were assessed using linear modelling with individual boar blocking and family relationship covariates

Several metabolites showed an abundance increase with age. Homoisovanillic acid and N-(1,3-Thiazol-2-yl)benzenesulfonamide abundance increased at 10 months, while succinic acid displayed increased abundance at 8.5 months compared to 7 months (adjusted *P* < 0.05).

**Fig. 5:**
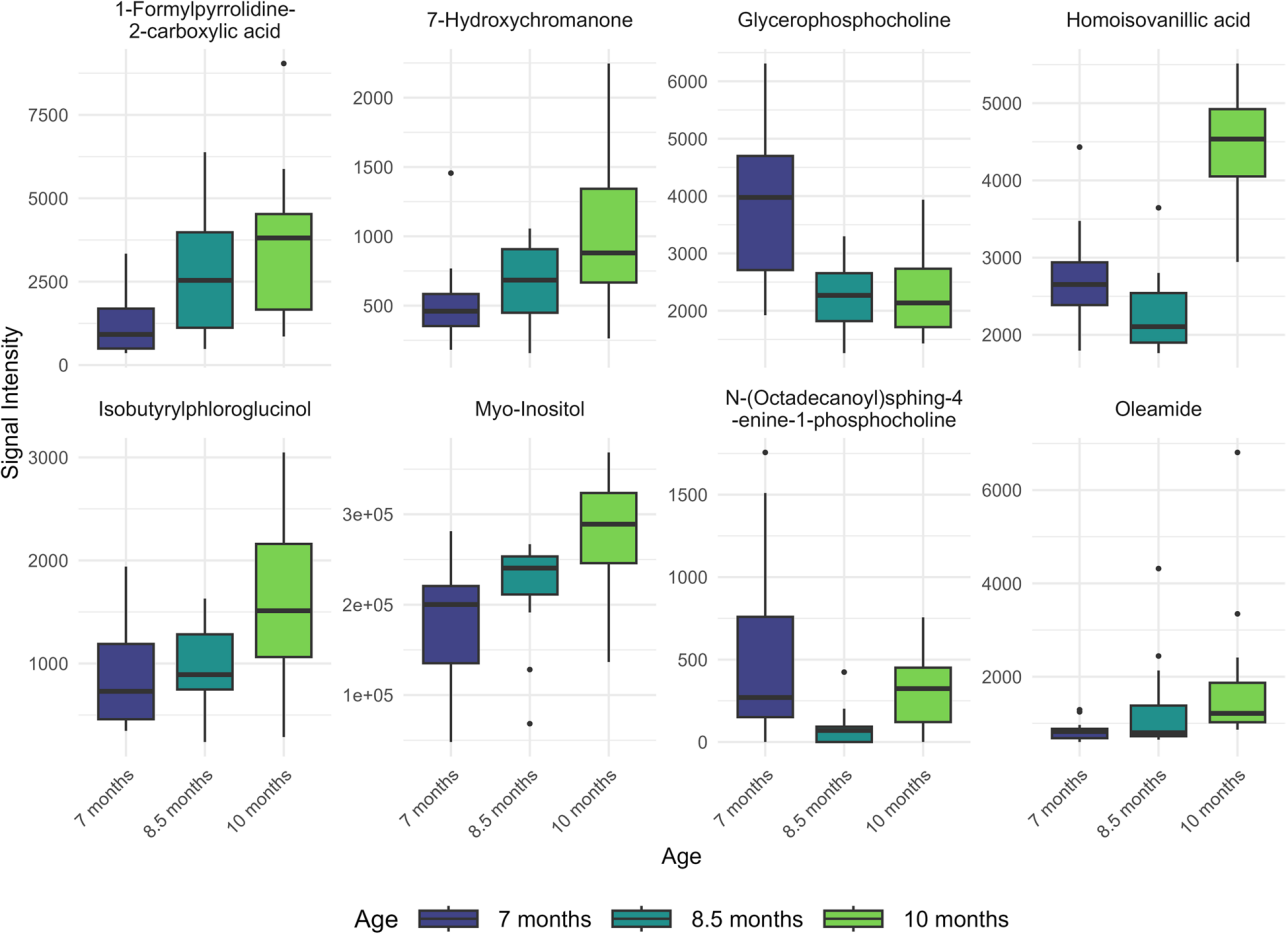
Longitudinal changes in boar seminal plasma metabolite abundance during sexual maturation. Box plots display the distribution of key metabolites across three ages in boar seminal plasma. The *x*-axis denotes age groups as 7 months, 8.5 months and 10 months, while the *y*-axis indicates the abundance of each metabolite. Semen samples were collected from 15 Duroc boars at three time points: initially when the animals were approximately 7 months old, followed by subsequent collections at 8.5 and 10 months. Seminal plasma was separated from spermatozoa, and metabolite abundances were measured using liquid chromatography-mass spectrometry (LC–MS) with HILIC and RP-Pos methods

Similarly, the longitudinal analysis of boar seminal plasma identified 17 annotated metabolites with significant temporal abundance changes across three ages (*P* < 0.05, Fig. 5, Table 3). 1-Formylpyrrolidine-2-carboxylic acid and myo-inositol exhibited the most statistically significant changes (adjusted *P* = 1.81 × 10^–4^), showing increased abundance at 10 months compared to 7 months. The abundance of 1-formylpyrrolidine-2-carboxylic acid also increased at 8.5 months (adjusted *P* < 0.05). Homoisovanillic acid likewise displayed elevated abundance at 10 months in comparison to both 8.5 months and 7 months (adjusted *P* < 0.05). Several metabolites showed an increase in abundance at 10 months relative to 7 months (adjusted *P* < 0.05), including 7-hydroxychromanone, oleamide, isobutyrylphloroglucinol, 4-O-β-galactopyranosyl-D-mannopyranose, and oleoyl ethylamide.

**Table 3:** List of annotated metabolites with significant abundance changes in boar seminal plasma across ages

Metabolite	Contrast	logFC	Fold change	SE	*t*-statistic	Adjusted *P* value
Glycerophosphocholine	10 M vs. 7 M	−1.31	2.48	0.30	−4.29	4.83 × 10^–4^
Glycerophosphocholine	8.5 M vs. 7 M	−0.96	1.94	0.30	−3.14	2.42 × 10^–2^
N-(Octadecanoyl)sphing-4-enine-1-phosphocholine	8.5 M vs. 7 M	−1.13	2.19	0.31	−3.65	1.23 × 10^–2^
Glyceric acid	10 M vs. 7 M	−1.06	2.09	0.32	−3.34	5.91 × 10^–3^
3-Indoleacetic acid	10 M vs. 7 M	−0.95	1.93	0.31	−3.05	1.42 × 10^–2^
3-Indoleacetic acid	8.5 M vs. 7 M	−0.93	1.91	0.31	−3.01	2.81 × 10^–2^
3'-Galactosyllactose	10 M vs. 7 M	−0.88	1.84	0.32	−2.76	3.17 × 10^–2^
4,4,7a-Trimethyl-3a,5,6,7-tetrahydro-3H-indene-1-carboxylic acid	10 M vs. 7 M	−0.88	1.84	0.31	−2.80	2.69 × 10^–2^
1-Formylpyrrolidine-2-carboxylic acid	10 M vs. 7 M	1.41	2.66	0.32	4.44	1.81 × 10^–4^
Myo-inositol	10 M vs. 7 M	1.39	2.62	0.32	4.38	1.81 × 10^–4^
Homoisovanillic acid	10 M vs. 7 M	1.38	2.60	0.30	4.58	2.8 × 10^–4^
Homoisovanillic acid	10 M vs. 8.5 M	1.35	2.55	0.30	4.48	4.28 × 10^–4^
7-Hydroxychromanone	10 M vs. 7 M	1.20	2.30	0.31	3.87	1.78 × 10^–3^
Oleamide	10 M vs. 7 M	1.15	2.21	0.31	3.71	2.41 × 10^–3^
Isobutyrylphloroglucinol	10 M vs. 7 M	1.12	2.17	0.31	3.59	3.07 × 10^–3^
4-O-β-Galactopyranosyl-D-mannopyranose	10 M vs. 7 M	1.11	2.16	0.32	3.50	4.41 × 10^–3^
1-Formylpyrrolidine-2-carboxylic acid	8.5 M vs. 7 M	1.10	2.14	0.32	3.45	1.57 × 10^–2^
Oleoyl ethylamide	10 M vs. 7 M	1.04	2.05	0.32	3.28	7.75 × 10^–3^
5'-S-Methyl-5'-thioadenosine	8.5 M vs. 7 M	0.99	1.98	0.31	3.19	2.42 × 10^–2^
Methanesulfonic acid	10 M vs. 7 M	0.82	1.76	0.31	2.61	4.1 × 10^–2^

Fold changes are calculated as 2^logFC^. Ages correspond to 7 months (7 M), 8.5 months (8.5 M), and 10 months (10 M). SE stands for standard error. Semen samples were obtained from 15 Duroc boars at three time points: initially when the animals were approximately 7 months, followed by subsequent collections at 8.5 and 10 months. Seminal plasma was separated from spermatozoa, and liquid chromatography-mass spectrometry (LC–MS) with HILIC and RP-Pos methods was used to measure metabolite abundances in seminal plasma. Metabolite differences were assessed using linear modelling with individual boar blocking and family relationship covariates

Several metabolites exhibited a decrease in abundance at 10 months compared to 7 months (adjusted *P* < 0.05) in seminal plasma, including glycerophosphocholine, glyceric acid, and 3-indoleacetic acid. Glycerophosphocholine and 3-indoleacetic acid also demonstrated reduced abundance at 8.5 months relative to 7 months. Similarly, N-(octadecanoyl)sphing-4-enine-1-phosphocholine showed a decrease at 8.5 months in comparison to 7 months.

### Correlation of semen metabolites with age

Partial Spearman correlation analysis, adjusting for boar variability, confirmed strong relationships between age and four annotated metabolites in spermatozoa (Additional file 4: Table S4): L-glutamic acid (ρ = −0.58, FDR = 0.0009), decanoyl-L-carnitine (ρ = −0.45, FDR = 0.035), PC(O-16:0/22:6) (ρ = −0.41, FDR = 0.044) and N-(1,3-Thiazol-2-yl)benzenesulfonamide (ρ = 0.57, FDR = 0.0009). These aligned with linear model trends (e.g., L-glutamic acid’s abundance decreases in older ages of boars, whereas N-(1,3-Thiazol-2-yl)benzenesulfonamide’s abundance increases with age). Glycerophosphocholine and octanoylcarnitine showed negative correlation with age (ρ = −0.43, FDR = 0.035), and DL-indole-3-lactic acid showed positive correlation with age (ρ = 0.43, FDR = 0.035).

Likewise, in seminal plasma, the partial correlation analysis confirmed temporal associations for 12 annotated metabolites in seminal plasma (FDR < 0.05, Additional file 5: Table S5). Among these, homoisovanillic acid, 1-formylpyrrolidine-2-carboxylic acid, myo-inositol, 7-hydroxychromanone, oleamide, isobutyrylphloroglucinol, 4-O-β-galactopyranosyl-D-mannopyranose and oleoyl ethylamide showed significant positive correlation with age (ρ = 0.42–0.58, FDR < 0.05), aligning with their progressive accumulation in linear models. Glycerophosphocholine, glyceric acid and 3-indoleacetic acid showed significant negative correlation (ρ = −0.40 to −0.53, FDR < 0.05), consistent with sustained suppression in linear models, suggesting a decrease in abundance with increasing age. DL-indole-3-lactic acid emerged exclusively in correlation analysis with gradual reduction in abundance (ρ = −0.39, FDR < 0.05) with age.

### Ranking of semen metabolites

**Fig. 6:**
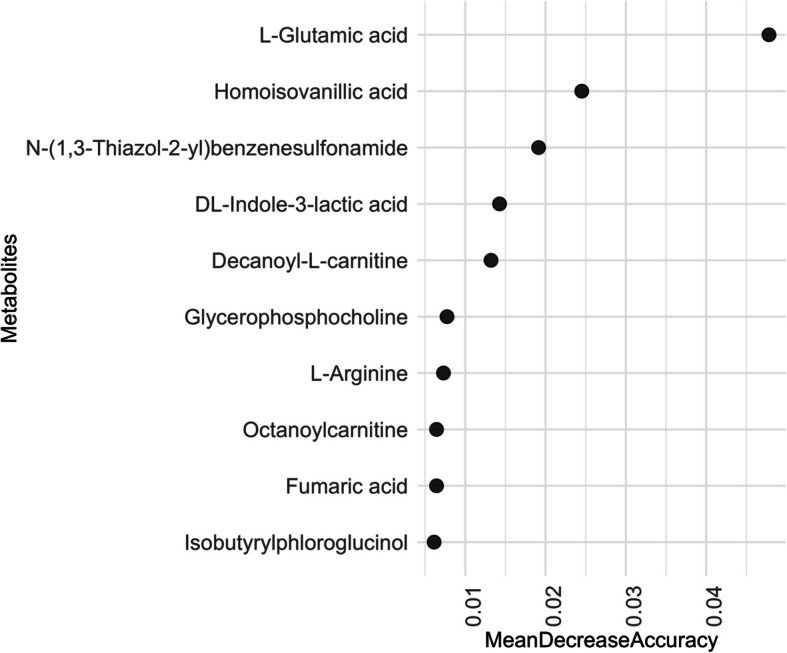
Top 10 metabolites distinguishing age groups in boar spermatozoa. Metabolites are ranked by mean decrease accuracy (*x*-axis) from random forest analysis modelling. The plot shows metabolites on the *y*-axis listed in descending order of their importance for age group classification. Semen samples were collected from 15 Duroc boars at three time points: initially when the animals were approximately 7 months old, followed by subsequent collections at 8.5 and 10 months. Spermatozoa were isolated from the seminal plasma, and metabolite abundances were measured using liquid chromatography-mass spectrometry (LC–MS) with HILIC and RP-Pos methods

In spermatozoa, random forest analysis with an average OOB error of 0.43 ranked L-glutamic acid (MDA = 0.048), homoisovanillic acid (MDA = 0.025), N-(1,3-Thiazol-2-yl)-benzenesulfonamide (MDA = 0.019), DL-indole-3-lactic acid (MDA = 0.014) and decanoyl-L-carnitine (MDA = 0.013) as the top five contributors (Fig. 6, Additional file 6: Table S6) to the predictive accuracy of the age group categories.

**Fig. 7:**
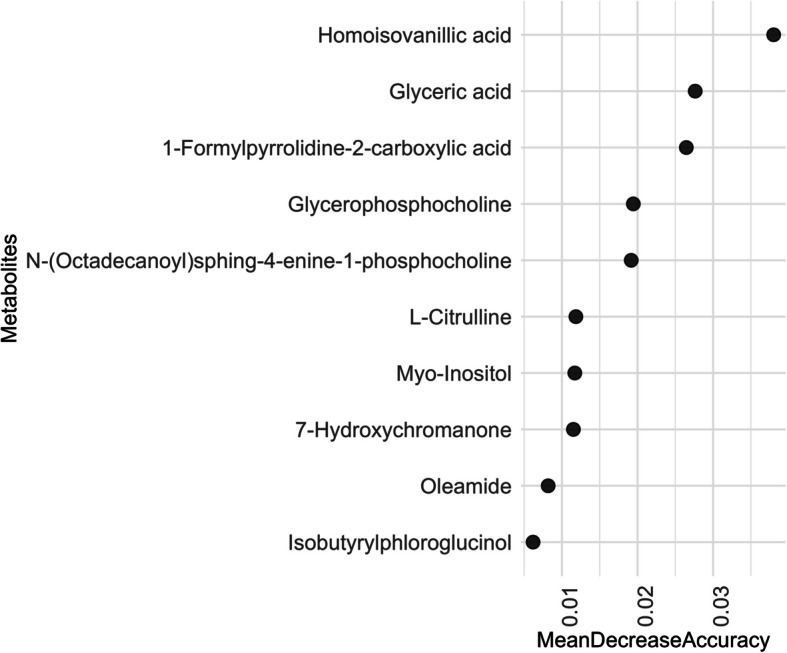
Top 10 metabolites distinguishing age groups in boar seminal plasma. Metabolites are ranked by mean decrease accuracy (*x*-axis) from random forest analysis modelling. The plot shows metabolites on the *y*-axis listed in descending order of their importance for age group classification. Semen samples were collected from 15 Duroc boars at three time points: initially when the animals were approximately 7 months old, followed by subsequent collections at 8.5 and 10 months. Seminal plasma was separated from spermatozoa, and metabolite abundances were measured using liquid chromatography-mass spectrometry (LC–MS) with HILIC and RP-Pos methods

Similarly, in seminal plasma, random forest analysis with an average OOB error of 0.47 ranked homoisovanillic acid (MDA = 0.064), 1-formylpyrrolidine-2-carboxylic acid (MDA = 0.025), glyceric acid (MDA = 0.025), myo-inositol (MDA = 0.020) and N-(octadecanoyl)sphing-4-enine-1-phosphocholine (MDA = 0.017) as the top five contributors (Fig. 7) to the predictive accuracy of the age group categories (Additional file 7: Table S7).

## Discussion

This study represents, to our knowledge, the first comprehensive untargeted metabolomic investigation of boar spermatozoa and seminal plasma across three distinct maturation timepoints. Following the boars longitudinally from puberty through sexual maturity, we have expanded our understanding of the metabolic landscape that highlights the physiological transformations underlying sexual maturity.

The annotated metabolite profile in boar semen was predominantly comprised of organic acids and derivatives, followed by lipids and lipid-like molecules, with amino acids and fatty acids dominating the composition. This compositional distribution aligns with earlier studies in bull sperm and seminal plasma [26, 30] and extends our previous findings on the amino acid and amine composition in boar semen [32]. These results support the established understanding of mammalian semen composition and its essential roles in sperm physiology as described by Brown-Woodman et al. [42]. For bulls, it has been reported that variations in fatty acid profiles, including myristic acid, polyunsaturated fatty acids (PUFAs), docosahexaenoic acid (DHA), omega-3 fatty acids, and cholesterol, correlate with differences in sperm motility, morphology, and concentration evident in comparative studies of young versus mature bulls [43].

Our multivariate analyses encompassing all detected features revealed maturation trajectories in the metabolomic landscape. The increased metabolomic variability observed in 7-month-old boars likely reflects the transitional metabolic state during maturation, where individual boars may be at different stages of sexual maturation. The ability of PLS-DA to distinguish between the youngest cohort and the two mature groups in both spermatozoa and seminal plasma suggests that there is a shift in metabolic profile during the transition from puberty to sexual maturity, even across the relatively modest six-week intervals examined in our study. These findings complement and extend our previous observations of age-dependent clustering in amino acid and amine profiles [32].

Pathway analysis in this study revealed three key metabolic pathways in spermatozoa underlying sexual maturation: porphyrin metabolism, taurine and hypotaurine metabolism, and glycerolipid metabolism. Porphyrin metabolism is primarily associated with heme biosynthesis, supporting cellular respiration and redox regulation, which are vital during sperm maturation due to high energy demands associated with motility [44].

The enrichment of taurine and hypotaurine metabolism pathways in our dataset aligns with their known abundance in male reproductive tissues, including testis and epididymis [45]. These compounds are particularly concentrated in the epididymis, where sperm maturation occurs [46]. Hypotaurine effectively scavenges reactive oxygen species (ROS), thus preventing lipid peroxidation and preserving membrane integrity, as shown in rabbit spermatozoa [47] and chicken sperm during cryopreservation [48]. It can also maintain mitochondrial integrity, inhibit DNA fragmentation, and promote spermatogenesis through effects on the hypothalamus-pituitary-testis axis [45, 48]. In taurine-supplemented boar semen during storage, it is reported that taurine improves motility and viability [49].

The glycerolipid metabolism pathways are involved in maintaining membrane integrity. Polyunsaturated fatty acids (PUFAs), particularly DHA, maintain membrane fluidity and support motility in boar sperm [50]. In mice, lipid remodelling in the epididymis, characterised by shifts in phosphatidylcholine (PC) acyl groups to DHA and docosapentaenoic acid, significantly enhances sperm motility and fertility​ [51], processes important for sperm maturation.

Likewise, pathway analysis of our seminal plasma data revealed significant enrichment of glutathione metabolism and nitrogen metabolism pathways during maturation. Glutathione and its associated enzymes, such as glutathione peroxidase (GPx), provide dual protection by scavenging H_2_O_2_ and regenerating oxidised molecules, thereby preserving membrane integrity and sperm motility [52, 53]. Moreover, the enrichment of nitrogen metabolism pathways in our seminal plasma analysis highlights the importance of nitrogen-containing metabolites such as arginine, proline, and citrulline, which contribute to nitric oxide production, ammonia detoxification, osmotic regulation, and antioxidant defence. These functions are critical for sperm survival under stress conditions [54]. Although direct evidence linking nitrogen metabolism to sexual maturation remains limited, the involvement of these metabolites provides an interesting basis for further exploration.

Homoisovanillic acid, a breakdown product of dopamine, emerged as a significant metabolite in both spermatozoa and seminal plasma in our study, suggesting fundamental roles in sexual maturity. It has been reported that dopamine activates male sexual behaviour, even in the absence of estrogen receptor α [55], governing both anticipatory (motivation) and consummatory (erection and copulation) phases of sexual behaviour [56]. A structurally related phenolic compound, vanillic acid, has been well documented for its antioxidative properties, such as scavenging free radicals, inhibiting lipid peroxidation, and activation of endogenous antioxidant enzymes, superoxide dismutase and catalase [57, 58]. This protective effect could be especially relevant in the epididymal environment, maintained by V-ATPase and other acid/base transporters, where maintaining the acidic environment is integral for sperm maturation [59, 60].

L-Glutamic acid declined with maturation in this study, consistent with our previous findings [32] and its reported roles in modulating sperm motility, chemotaxis, acrosome reaction, sperm development, and mitochondrial regulation [61–65]. Mechanistically, glutamate binds to N-methyl-D-aspartate receptors, causing calcium influx and magnesium release, which are vital for sperm motility [63] and the acrosome reaction required for oocyte penetration [64, 65]. Glutamate also enhances antioxidant defences, regulates inflammatory responses, and promotes spermatogonial cell proliferation [66, 67].

Decanoyl-L-carnitine, formed through esterification of L-carnitine with decanoic acid [68], was another important metabolite identified in boar sperm by this study. We observed declining decanoyl-L-carnitine abundance with age, suggesting regulatory adjustments in fatty acid metabolism during this critical maturation window. It has been reported that L-carnitine improves progressive and total sperm motility in Duroc boars [69] and Simmental bulls [70], while protecting maturing sperm from oxidative stress by boosting antioxidant enzyme activities. In addition, it supports energy metabolism by generating ATP via transport of long-chain fatty acids into mitochondria for β-oxidation [71, 72]. N-(1,3-Thiazol-2-yl)benzenesulfonamide, a sulfathiazole derivative belonging to the sulfonamide class [73], showed increasing abundance with age in our study. The involvement of sulfonamide-based metabolites in diverse biological activities, including modulation of oxidative stress and cellular signalling pathways [74, 75], highlights their possible influence on spermatogenesis and sperm maturation, which warrants further investigation.

Our seminal plasma metabolome analyses revealed distinctive age-dependent signatures. 1-Formylpyrroline-2-carboxylic acid showed a significant elevation in mature boars (10 months) in our study, aligning with its known antioxidant properties and effects on cellular redox states [76, 77], suggesting a protective role during sperm maturation. Similarly, 7-hydroxychromanone accumulated steadily with advancing age, bringing potent ROS scavenging and lipid peroxidation​ inhibition that likely preserve sperm viability, motility and DNA integrity [78, 79]. Oleamide showed a similar increasing abundance in our study, most likely contributing to anti-inflammatory and antioxidant functions​ [80, 81]. This aligns with the broader role of fatty acid derivatives in spermatogenesis, sperm maturation and improving sperm quality, as highlighted in previous studies​ [82].

Myo-inositol, a naturally occurring cyclohexane hexol belonging to the vitamin B complex (B8), had an increasing abundance as boars mature in our study. It is interesting to note that previous studies have reported an association of myo-inositol with increased sperm motility, acrosome integrity, testosterone levels, maintenance of DNA integrity of sperm and fertilisation ability [83, 84]. It is noteworthy that motility also increased with age in our study, which implies role of myo-inositol in motility regulation. Its regulatory effect, earlier reported on intracellular calcium levels, further supports mitochondrial function and ATP production, essential for sperm capacitation and maturation [85, 86]. Glycerophosphocholine is predominantly synthesised in the epididymis under androgenic control, and glyceric acid displayed a decline with advancing age in our analysis. Glycerophosphocholine’s roles in osmoregulation and membrane composition [87, 88] combined with glyceric acid’s involvement in glycolysis [89] and mitochondrial metabolism activation and reduction of systemic inflammation in humans [90] suggest their decline represents optimisation of conditions for maturing spermatozoa. N-(Octadecanoyl)sphing-4-enine-1-phosphocholine, a sphingolipid component of sphingomyelin, showed a significant decline at 8.5 months compared to 7 months. Sphingomyelins contribute to membrane stability and fluidity, supporting critical functions including lipid raft formation and signal transduction essential for capacitation and the acrosome reaction [91, 92]. Their metabolism generates bioactive compounds like ceramides and sphingosine-1-phosphate that regulate apoptosis, lipid remodelling, and gametogenesis, helping maintain membrane integrity and enabling the structural changes necessary for fertilisation ​​[91, 93]. Additionally, sphingolipids facilitate cholesterol efflux, enhancing membrane fluidity and protein interactions critical for maturation [92]​​, while contributing antioxidant protection against oxidative damage​​ [91].

In our study, we selected three age timepoints: 7 months (puberty with onset of mounting behaviour and first ejaculates), 8.5 months (the industry standard for commercial semen collection), and 10 months (sexual maturity with consistent sperm production). This 6-week interval design effectively captured the metabolic changes during this critical puberty to sexual maturation window. The longitudinal design following 15 Duroc boars eliminated genetic variability and captured true metabolic changes with sexual maturation rather than inter-individual differences. However, our modest sample size and single-breed focus limit broader applicability to other genetic lines. In addition, the challenge of metabolite annotation persisted, leaving a considerable number of features (over 4,300) unidentified, potentially concealing valuable biological insights that could prove useful in future studies. Improved metabolite annotation would help unlock this hidden information, providing a deeper understanding of the underlying biological processes.

Our comprehensive metabolomics investigation has revealed complex metabolic changes in boar spermatozoa and seminal plasma during the transition from puberty to sexual maturity. The identified metabolites and pathways contributing to membrane integrity, oxidative protection, and energy metabolism provide fundamental insights into reproductive development. This foundational characterisation enables future development of biomarker-based screening protocols for the early identification of younger boars ready for breeding, reducing generation intervals and accelerating genetic progress in commercial operations. Our findings also provide a knowledge base for developing targeted nutritional interventions to potentially shift metabolite concentrations in young males toward a mature metabolic profile. Furthermore, these metabolites can serve as candidates for semen extender supplementation for young males.

However, validation in larger, more diverse populations will be essential, along with targeted analyses including focused lipidomics and functional assays to clarify the biological roles of these metabolites. Additionally, investigating how these metabolic signatures correlate with fertility outcomes will strengthen their practical relevance to reproductive biotechnology and commercial pig breeding programs.

## Conclusions

Our results reveal subtle yet important shifts in the metabolomic composition in boar sperm and seminal plasma as they grow from puberty to sexual maturity. We identified numerous metabolites and pathways important in distinguishing these age groups during sexual maturation. This insight offers a valuable understanding of the evolving metabolome, which can serve as the foundation for future research and potential applications in the field of reproductive biology, especially for pig breeding companies interested in incorporating younger animals in the breeding program.

## Supplementary Information

Additional file 1: Table S1: Overview of annotated metabolites in boar semen, including their respective numbers with their chemical classes and superclasses. Note: The metabolites are extracted using global metabolomics with LC–MS platform, including HILIC and RP-Pos methods.

Additional file 2: Table S2: Pathway enrichment analysis results of all features in boar spermatozoa. Note: The table includes pathway’s name, total hits, significant hits, expected values, Fisher’s exact test *P*-values, and enrichment factors.

Additional file 3: Table S3: Pathway enrichment analysis results of all features in boar seminal plasma. Note: The table includes pathway’s name, total hits, significant hits, expected values, Fisher’s exact test *P*-values, and enrichment factors.

Additional file 4: Table S4: Partial Spearman correlation analysis of annotated metabolites with age in boar spermatozoa. Note: The table shows partial Spearman correlation coefficients, t-statistics, and false discovery rateadjustments.

Additional file 5: Table S5: Partial Spearman correlation analysis of annotated metabolites with age in boar seminal plasma. Note: The table shows Spearman correlation coefficients, t-statistics, and false discovery rateadjustments.

Additional file 6: Table S6: Mean decrease accuracy values of annotated metabolites in boar spermatozoa. Note: It is generated from a random forest analysis.

Additional file 7: Table S7: Mean decrease accuracy values of annotated metabolites in boar seminal plasma. Note: It is generated from a random forest analysis.

## Data Availability

The datasets used and/or analysed during the current study are available from the corresponding author on reasonable request.

## Abbreviations

AI: Artificial insemination
DHA: Docosahexanoic acid
FDR: False discovery rate
HILIC: Hydrophilic interaction liquid chromatography
LC–MS: Liquid chromatography–mass spectrometry
MDA: Mean decrease accuracy
OOB: Out-of-bag
PC: Principal component
PCA: Principal component analysis
PERMANOVA: Permutational multivariate analysis of variance
PLS-DA: Partial least squares discriminant analysis
PUFA: Polyunsaturated fatty acid
ROS: Reactive oxygen species
RP-Pos: Reverse-phase (positive ionisation mode)
