# Supplementary material for: Untargeted metabolomics reveals changes in boar sperm and seminal plasma metabolites associated with sexual maturity
Source: J Anim Sci Biotechnol. 2025 Sep 3;16:123. doi: 10.1186/s40104-025-01258-x (PMC12406428; doi:10.1186/s40104-025-01258-x)
Supplement: Supplementary file 1 — Additional file 1: Table S1: Overview of annotated metabolites in boar semen, including their respective numbers with their chemical classes and superclasses. Note: The metabolites are extracted using global metabolomics with LC–MS platform, including HILIC and RP-Pos methods. [file 40104_2025_1258_MOESM1_ESM.docx]

Table S1: Overview of annotated metabolites in boar semen, including their respective numbers with their chemical classes and superclasses. The metabolites are extracted using global metabolomics with LC-MS platform, including HILIC and RP-Pos methods.

| S.N | Metabolites | | Count |
| --- | --- | --- | --- |
| **1.** | **Organic acids and derivatives** | | **28** |
| Amino acids, peptides, and analogues | | | 13 |
| Carboxylic acid derivatives | | | 1 |
| Carboxylic acids | | | 3 |
| Dicarboxylic acids and derivatives | | | 2 |
| Gamma-keto acids and derivatives | | | 1 |
| Organosulfonic acids and derivatives | | | 2 |
| Phosphate esters | | | 2 |
| Tricarboxylic acids and derivatives | | | 3 |
| Urea | | | 1 |
| **2.** | **Lipids and Lipid-like molecules** | | **26** |
| Fatty acid esters | | | 10 |
| Fatty acids and conjugates | | | 2 |
| Fatty amides | | | 4 |
| Glycerophosphocholines | | | 6 |
| Monoradylglycerols | | | 2 |
| Phosphosphingolipids | | | 2 |
| **3.** | | **Organic oxygen compounds** | **13** |
| Alcohols and polyols | | | 1 |
| Carbohydrates and carbohydrate conjugates | | | 4 |
| Carbonyl compounds | | | 2 |
| Ethers | | | 6 |
| **4.** | | **Benzenoids** | **8** |
| Benzenesulfonamides | | | 1 |
| Benzoic acids and derivatives | | | 6 |
| Methoxyphenols | | | 1 |
| **5.** | | **Organoheterocyclic compounds** | **7** |
| Benzofuranones | | | 1 |
| Caprolactams | | | 1 |
| Indolyl carboxylic acids and derivatives | | | 3 |
| Morpholines | | | 1 |
| Quinolones and derivatives | | | 1 |
| **6.** | | **Organic nitrogen compounds** | **3** |
| Amines | | | 3 |
| **7.** | | **Nucleosides, nucleotides and analogues** | **2** |
| 5'-deoxy 5'-thionucleosides | | | 1 |
| Purine nucleosides | | | 1 |
| **8.** | | **Phenylpropanoids and polyketides** | **2** |
| 3,4-dihydrocoumarins | | | 1 |
| Cinnamyl alcohols | | | 1 |
| **9.** | | **Alkaloids and derivatives** | **1** |
| Tropane alkaloids | | | 1 |
| **10.** | | **Organometallic compounds** | **1** |
| Organosilicon compounds | | | 1 |
| **11.** | | **Organosulfur compounds** | **1** |
| Sulfones | | | 1 |
