# Supplementary material for: Untargeted metabolomics reveals changes in boar sperm and seminal plasma metabolites associated with sexual maturity
Source: J Anim Sci Biotechnol. 2025 Sep 3;16:123. doi: 10.1186/s40104-025-01258-x (PMC12406428; doi:10.1186/s40104-025-01258-x)
Supplement: Supplementary file 2 — Additional file 2: Table S2: Pathway enrichment analysis results of all features in boar spermatozoa. Note: The table includes pathway’s name, total hits, significant hits, expected values, Fisher’s exact test P-values, and enrichment factors. [file 40104_2025_1258_MOESM2_ESM.docx]

Table S2: Pathway enrichment analysis results of all features in boar spermatozoa. It includes pathway’s name, total hits, significant hits, expected values, Fisher’s exact test *P*-values, and enrichment factors.

| S.N. | Pathway | Pathway total | Hits total | Hits sig. | Expected | *P* (Fisher) | Enrichment factor |
| --- | --- | --- | --- | --- | --- | --- | --- |
| 1. | Porphyrin metabolism | 31 | 2 | 2 | 0.32 | 0.002 | 6.25 |
| 2. | Taurine and hypotaurine metabolism | 8 | 2 | 2 | 0.16 | 0.006 | 12.50 |
| 3. | Glycerolipid metabolism | 16 | 5 | 4 | 1.36 | 0.006 | 2.94 |
| 4. | Glutathione metabolism | 28 | 3 | 1 | 0.40 | 0.05 | 2.50 |
| 5. | Glycerophospholipid metabolism | 36 | 3 | 2 | 0.40 | 0.05 | 5.00 |
| 6. | Nitrogen metabolism | 6 | 2 | 1 | 0.40 | 0.05 | 2.50 |
| 7. | Primary bile acid biosynthesis | 46 | 1 | 1 | 0.08 | 0.08 | 12.50 |
| 8. | D-Amino acid metabolism | 15 | 14 | 3 | 1.12 | 0.09 | 2.68 |
| 9. | Histidine metabolism | 16 | 5 | 1 | 0.56 | 0.10 | 1.79 |
| 10. | Ether lipid metabolism | 20 | 1 | 1 | 0.16 | 0.15 | 6.25 |
| 11. | Tyrosine metabolism | 42 | 2 | 2 | 0.19 | 0.19 | 10.33 |
| 12. | Drug metabolism - cytochrome P450 | 27 | 1 | 1 | 0.24 | 0.22 | 4.17 |
| 13. | Fructose and mannose metabolism | 20 | 11 | 5 | 1.68 | 0.23 | 2.98 |
| 14. | Terpenoid backbone biosynthesis | 18 | 3 | 1 | 0.32 | 0.29 | 3.13 |
| 15. | Steroid biosynthesis | 41 | 14 | 2 | 1.06 | 0.29 | 1.88 |
| 16. | Arginine and proline metabolism | 36 | 15 | 5 | 1.92 | 0.30 | 2.60 |
| 17. | Glycolysis / Gluconeogenesis | 26 | 9 | 3 | 2.00 | 0.32 | 1.50 |
| 18. | Butanoate metabolism | 15 | 8 | 2 | 2.00 | 0.32 | 1.00 |
| 19. | Fatty acid biosynthesis | 47 | 3 | 1 | 0.39 | 0.34 | 2.58 |
| 20. | Arginine biosynthesis | 14 | 8 | 1 | 1.20 | 0.34 | 0.83 |
| 21. | Glyoxylate and dicarboxylate metabolism | 32 | 13 | 2 | 3.04 | 0.36 | 0.66 |
| 22. | Propanoate metabolism | 22 | 8 | 2 | 2.16 | 0.37 | 0.93 |
| 23. | Caffeine metabolism | 12 | 2 | 2 | 0.48 | 0.40 | 4.17 |
| 24. | beta-Alanine metabolism | 21 | 7 | 1 | 1.52 | 0.46 | 0.66 |
| 25. | Steroid hormone biosynthesis | 78 | 8 | 2 | 0.58 | 0.47 | 3.44 |
| 26. | Glycine, serine and threonine metabolism | 34 | 15 | 2 | 2.80 | 0.55 | 0.71 |
| 27. | Pentose phosphate pathway | 23 | 15 | 1 | 1.84 | 0.57 | 0.54 |
| 28. | Terpenoid backbone biosynthesis | 18 | 2 | 1 | 0.77 | 0.58 | 1.29 |
| 29. | Pyruvate metabolism | 23 | 8 | 2 | 1.92 | 0.60 | 1.04 |
| 30. | Fatty acid biosynthesis | 47 | 4 | 1 | 0.96 | 0.64 | 1.04 |
| 31. | Starch and sucrose metabolism | 18 | 11 | 2 | 0.96 | 0.64 | 2.08 |
| 32. | Ascorbate and aldarate metabolism | 10 | 6 | 3 | 2.16 | 0.67 | 1.39 |
| 33. | Alanine, aspartate and glutamate metabolism | 28 | 16 | 2 | 3.36 | 0.69 | 0.60 |
| 34. | Galactose metabolism | 27 | 21 | 7 | 1.28 | 0.75 | 5.47 |
| 35. | Valine, leucine and isoleucine degradation | 40 | 13 | 1 | 1.36 | 0.77 | 0.74 |
| 36. | Inositol phosphate metabolism | 29 | 5 | 1 | 1.36 | 0.77 | 0.74 |
| 37. | Cysteine and methionine metabolism | 33 | 6 | 1 | 1.60 | 0.83 | 0.63 |
| 38. | Amino sugar and nucleotide sugar metabolism | 42 | 17 | 4 | 1.76 | 0.85 | 2.27 |
| 39. | Citrate cycle (TCA cycle) | 20 | 9 | 1 | 2.48 | 0.94 | 0.40 |
