# Supplementary material for: Untargeted metabolomics reveals changes in boar sperm and seminal plasma metabolites associated with sexual maturity
Source: J Anim Sci Biotechnol. 2025 Sep 3;16:123. doi: 10.1186/s40104-025-01258-x (PMC12406428; doi:10.1186/s40104-025-01258-x)
Supplement: Supplementary file 3 — Additional file 3: Table S3: Pathway enrichment analysis results of all features in boar seminal plasma. Note: The table includes pathway’s name, total hits, significant hits, expected values, Fisher’s exact test P-values, and enrichment factors. [file 40104_2025_1258_MOESM3_ESM.docx]

Table S3: Pathway enrichment analysis results of all features in boar seminal plasma. It includes pathway’s name, total hits, significant hits, expected values, Fisher’s exact test *P*-values, and enrichment factors.

| S.N. | Pathway | Pathway total | Hits total | Hits sig. | Expected | *P* (Fisher) | Enrichment factor |
| --- | --- | --- | --- | --- | --- | --- | --- |
| 1. | Glutathione metabolism | 28 | 3 | 2 | 0.78 | 0.03 | 2.57 |
| 2. | Nitrogen metabolism | 6 | 2 | 2 | 0.78 | 0.03 | 2.57 |
| 3. | Butanoate metabolism | 15 | 8 | 4 | 3.89 | 0.07 | 1.03 |
| 4. | Citrate cycle (TCA cycle) | 20 | 9 | 6 | 4.82 | 0.08 | 1.24 |
| 5. | Alanine, aspartate and glutamate metabolism | 28 | 16 | 6 | 6.53 | 0.08 | 0.92 |
| 6. | Glyoxylate and dicarboxylate metabolism | 32 | 13 | 7 | 5.91 | 0.10 | 1.18 |
| 7. | Porphyrin metabolism | 31 | 2 | 1 | 0.62 | 0.12 | 1.61 |
| 8. | D-Amino acid metabolism | 15 | 14 | 4 | 2.18 | 0.16 | 1.84 |
| 9. | Glycerophospholipid metabolism | 36 | 3 | 2 | 0.78 | 0.17 | 2.57 |
| 10. | Arginine biosynthesis | 14 | 8 | 3 | 2.33 | 0.19 | 1.29 |
| 11. | Ascorbate and aldarate metabolism | 10 | 6 | 6 | 4.20 | 0.22 | 1.43 |
| 12. | Glycerolipid metabolism | 16 | 5 | 4 | 2.64 | 0.26 | 1.51 |
| 13. | Taurine and hypotaurine metabolism | 8 | 2 | 1 | 0.31 | 0.29 | 3.21 |
| 14. | Ether lipid metabolism | 20 | 1 | 1 | 0.31 | 0.29 | 3.21 |
| 15. | Histidine metabolism | 16 | 5 | 1 | 1.09 | 0.30 | 0.92 |
| 16. | Drug metabolism - cytochrome P450 | 27 | 1 | 1 | 0.47 | 0.40 | 2.14 |
| 17. | Propanoate metabolism | 22 | 8 | 3 | 4.20 | 0.41 | 0.71 |
| 18. | Lysine degradation | 30 | 2 | 1 | 0.62 | 0.49 | 1.61 |
| 19. | Terpenoid backbone biosynthesis | 18 | 3 | 1 | 0.62 | 0.49 | 1.61 |
| 20. | Pentose phosphate pathway | 23 | 15 | 3 | 3.58 | 0.50 | 0.84 |
| 21. | Valine, leucine and isoleucine degradation | 40 | 13 | 1 | 2.64 | 0.51 | 0.38 |
| 22. | Arginine and proline metabolism | 36 | 15 | 4 | 3.73 | 0.53 | 1.07 |
| 23. | Pyrimidine metabolism | 38 | 8 | 3 | 1.71 | 0.53 | 1.75 |
| 24. | Lipoic acid metabolism | 27 | 3 | 2 | 1.71 | 0.53 | 1.17 |
| 25. | Fatty acid elongation | 39 | 2 | 1 | 0.78 | 0.57 | 1.29 |
| 26. | Fatty acid biosynthesis | 47 | 4 | 2 | 1.87 | 0.58 | 1.07 |
| 27. | Fatty acid degradation | 39 | 3 | 1 | 0.93 | 0.64 | 1.07 |
| 28. | Caffeine metabolism | 12 | 2 | 2 | 0.93 | 0.64 | 2.14 |
| 29. | Fructose and mannose metabolism | 20 | 11 | 6 | 3.27 | 0.67 | 1.84 |
| 30. | Purine metabolism | 71 | 12 | 2 | 2.18 | 0.67 | 0.92 |
| 31. | Biosynthesis of unsaturated fatty acids | 36 | 3 | 1 | 1.09 | 0.70 | 0.92 |
| 32. | Amino sugar and nucleotide sugar metabolism | 42 | 17 | 7 | 3.42 | 0.70 | 2.05 |
| 33. | Galactose metabolism | 27 | 21 | 9 | 2.49 | 0.75 | 3.62 |
| 34. | Pentose and glucuronate interconversions | 19 | 13 | 2 | 2.64 | 0.78 | 0.76 |
| 35. | Inositol phosphate metabolism | 29 | 5 | 2 | 2.64 | 0.78 | 0.76 |
| 36. | Glycolysis / Gluconeogenesis | 26 | 9 | 4 | 3.89 | 0.79 | 1.03 |
| 37. | beta-Alanine metabolism | 21 | 7 | 2 | 2.96 | 0.83 | 0.68 |
| 38. | Starch and sucrose metabolism | 18 | 11 | 2 | 1.87 | 0.88 | 1.07 |
| 39. | Pyruvate metabolism | 23 | 8 | 3 | 3.73 | 0.92 | 0.80 |
| 40. | Glycine, serine and threonine metabolism | 34 | 15 | 2 | 5.44 | 0.94 | 0.37 |
| 41. | Cysteine and methionine metabolism | 33 | 6 | 1 | 3.11 | 0.97 | 0.32 |
| 42. | Tyrosine metabolism | 42 | 13 | 1 | 3.89 | 0.99 | 0.26 |
