# Supplementary material for: Untargeted metabolomics reveals changes in boar sperm and seminal plasma metabolites associated with sexual maturity
Source: J Anim Sci Biotechnol. 2025 Sep 3;16:123. doi: 10.1186/s40104-025-01258-x (PMC12406428; doi:10.1186/s40104-025-01258-x)
Supplement: Supplementary file 4 — Additional file 4: Table S4: Partial Spearman correlation analysis of annotated metabolites with age in boar spermatozoa. Note: The table shows partial Spearman correlation coefficients, t-statistics, and false discovery rateadjustments. [file 40104_2025_1258_MOESM4_ESM.docx]

Table S4: Partial Spearman correlation analysis of annotated metabolites with age in boar spermatozoa. It shows partial Spearman correlation coefficients, t-statistics, and false discovery rate (FDR) adjustments.

| Metabolites | Correlation coefficient | t-statistics | FDR |
| --- | --- | --- | --- |
| N-(1,3-Thiazol-2-yl)benzenesulfonamide | 0.565 | 4.440 | 8.76 × 10^-4^ |
| L-Glutamic acid | -0.576 | -4.561 | 8.76 × 10^-4^ |
| DL-Indole-3-lactic acid | 0.431 | 3.097 | 0.035 |
| Octanoylcarnitine | -0.430 | -3.089 | 0.035 |
| Glycerophosphocholine | -0.432 | -3.106 | 0.035 |
| Decanoyl-L-carnitine | -0.452 | -3.287 | 0.035 |
| PC(O-16:0/22:6) | -0.411 | -2.922 | 0.044 |
| Homoisovanillic acid | 0.394 | 2.780 | 0.053 |
| Succinic acid | 0.373 | 2.604 | 0.086 |
| 3'-Galactosyllactose | -0.386 | -2.712 | 0.086 |
| N-(Octadecanoyl)sphing-4-enine-1-phosphocholine | -0.356 | -2.471 | 0.098 |
| Myristoyl-L-carnitine | -0.344 | -2.376 | 0.108 |
| L-Aspartic acid | 0.335 | 2.307 | 0.141 |
| Palmitoyl sphingomyelin | -0.305 | -2.076 | 0.191 |
| 1-Myristoyl-sn-glycero-3-phosphocholine | -0.292 | -1.976 | 0.214 |
| L-Arginine | 0.289 | 1.956 | 0.225 |
| Guanosine | -0.288 | -1.947 | 0.225 |
| 1-(1Z-Octadecenyl)-2-(5Z,8Z,11Z,14Z-eicosatetraenoyl)-sn-glycero-3-phosphocholine | -0.281 | -1.896 | 0.230 |
| 1-Palmitoylglycerol | 0.266 | 1.792 | 0.261 |
| Lauroyl-L-carnitine | -0.261 | -1.750 | 0.262 |
| Isocitric acid | 0.268 | 1.802 | 0.265 |
| 4-Formyl-2-hydroxybenzoic acid | -0.253 | -1.697 | 0.271 |
| 7-Hydroxychromanone | -0.242 | -1.613 | 0.297 |
| 1-Formylpyrrolidine-2-carboxylic acid | 0.243 | 1.625 | 0.302 |
| Creatine | -0.246 | -1.642 | 0.302 |
| 8-Azabicyclo[3.2.1]octan-3-ol | -0.230 | -1.535 | 0.304 |
| 1-Hexadecyl-sn-glycero-3-phosphocholine | -0.231 | -1.540 | 0.304 |
| 3-Oxocyclobutanecarboxylic acid | -0.232 | -1.545 | 0.319 |
| Cyclo(leucylprolyl) | -0.214 | -1.420 | 0.353 |
| L-Serine | -0.214 | -1.419 | 0.367 |
| Isobutyrylphloroglucinol | -0.198 | -1.312 | 0.384 |
| 1-Palmitoyl-sn-glycero-3-phosphocholine | -0.202 | -1.335 | 0.384 |
| L-Citrulline | 0.202 | 1.336 | 0.392 |
| Hypaphorine | -0.181 | -1.198 | 0.422 |
| Erucamide | -0.183 | -1.203 | 0.422 |
| 2,3-Dihydroxypropyl octadecanoate | 0.162 | 1.064 | 0.465 |
| L-Carnitine | 0.160 | 1.054 | 0.465 |
| Hexanoyl-L-carnitine | -0.166 | -1.089 | 0.465 |
| DL-Phenylalanine | 0.168 | 1.102 | 0.534 |
| 5'-S-Methyl-5'-thioadenosine | 0.134 | 0.877 | 0.537 |
| 4,4,7a-Trimethyl-3a,5,6,7-tetrahydro-3H-indene-1-carboxylic acid | -0.135 | -0.886 | 0.537 |
| Oleoyl ethylamide | -0.140 | -0.919 | 0.537 |
| Fumaric acid | 0.147 | 0.963 | 0.614 |
| Methyl 1H-indol-3-ylacetate | 0.113 | 0.735 | 0.628 |
| 4-O-.beta.-Galactopyranosyl-D-mannopyranose | 0.137 | 0.899 | 0.631 |
| Methanesulfonic acid | -0.107 | -0.694 | 0.639 |
| Caffeoyl alcohol | 0.123 | 0.806 | 0.675 |
| 3-Benzylhexahydropyrrolo[1,2-a]pyrazine-1,4-dione | 0.088 | 0.570 | 0.697 |
| Oleamide | -0.089 | -0.581 | 0.697 |
| Urea | 0.082 | 0.536 | 0.703 |
| Itaconic acid | 0.086 | 0.559 | 0.745 |
| D-Fructose | -0.086 | -0.559 | 0.745 |
| 2-Oxopentanedioic acid | -0.095 | -0.617 | 0.745 |
| Taurine | -0.104 | -0.678 | 0.745 |
| L-Threonine | 0.077 | 0.499 | 0.761 |
| Valproic acid | 0.056 | 0.363 | 0.824 |
| Quinolin-2-ol | 0.044 | 0.283 | 0.849 |
| 3-Indoleacetic acid | 0.038 | 0.248 | 0.849 |
| Acetyl-L-carnitine | -0.041 | -0.266 | 0.849 |
| Heptadecasphing-4-enine | 0.026 | 0.170 | 0.871 |
| Isovaleryl-L-carnitine | 0.025 | 0.163 | 0.871 |
| Myo-Inositol | 0.044 | 0.286 | 0.895 |
| Glyceric acid | -0.040 | -0.261 | 0.895 |
| D-Aspartic acid | 0.027 | 0.177 | 0.927 |
| Trans-Aconitic acid | 0.021 | 0.136 | 0.927 |
| Citric acid | -0.011 | -0.069 | 0.946 |
