# Supplementary material for: Untargeted metabolomics reveals changes in boar sperm and seminal plasma metabolites associated with sexual maturity
Source: J Anim Sci Biotechnol. 2025 Sep 3;16:123. doi: 10.1186/s40104-025-01258-x (PMC12406428; doi:10.1186/s40104-025-01258-x)
Supplement: Supplementary file 5 — Additional file 5: Table S5: Partial Spearman correlation analysis of annotated metabolites with age in boar seminal plasma. Note: The table shows Spearman correlation coefficients, t-statistics, and false discovery rateadjustments. [file 40104_2025_1258_MOESM5_ESM.docx]

Table S5: Partial Spearman correlation analysis of annotated metabolites with age in boar seminal plasma. It shows Spearman correlation coefficients, t-statistics, and false discovery rate (FDR) adjustments.

| Metabolites | Correlation coefficient | t-statistics | FDR |
| --- | --- | --- | --- |
| Homoisovanillic acid | 0.581 | 4.630 | 0.001 |
| 1-Formylpyrrolidine-2-carboxylic acid | 0.555 | 4.326 | 0.001 |
| Myo-Inositol | 0.551 | 4.284 | 0.001 |
| Glyceric acid | -0.528 | -4.021 | 0.002 |
| Oleamide | 0.532 | 4.077 | 0.003 |
| 7-Hydroxychromanone | 0.524 | 3.984 | 0.003 |
| Glycerophosphocholine | -0.531 | -4.06 | 0.003 |
| Isobutyrylphloroglucinol | 0.508 | 3.817 | 0.003 |
| 4-O-.beta.-Galactopyranosyl-D-mannopyranose | 0.450 | 3.268 | 0.015 |
| Oleoyl ethylamide | 0.425 | 3.047 | 0.026 |
| 3-Indoleacetic acid | -0.406 | -2.878 | 0.035 |
| DL-Indole-3-lactic acid | -0.388 | -2.731 | 0.045 |
| Hypaphorine | -0.327 | -2.243 | 0.131 |
| 4,4,7a-Trimethyl-3a,5,6,7-tetrahydro-3H-indene-1-carboxylic acid | -0.302 | -2.052 | 0.181 |
| 3'-Galactosyllactose | -0.318 | -2.171 | 0.193 |
| Valproic acid | 0.279 | 1.883 | 0.236 |
| N-(1,3-Thiazol-2-yl)benzenesulfonamide | -0.288 | -1.947 | 0.262 |
| Decanoyl-L-carnitine | -0.261 | -1.754 | 0.282 |
| Itaconic acid | -0.269 | -1.810 | 0.299 |
| Heptadecasphing-4-enine | 0.239 | 1.594 | 0.355 |
| Isovaleryl-L-carnitine | -0.232 | -1.544 | 0.363 |
| L-Glutamic acid | -0.246 | -1.645 | 0.363 |
| DL-Phenylalanine | 0.221 | 1.469 | 0.448 |
| 5'-S-Methyl-5'-thioadenosine | 0.196 | 1.293 | 0.451 |
| 4-Formyl-2-hydroxybenzoic acid | 0.190 | 1.252 | 0.451 |
| Methanesulfonic acid | 0.189 | 1.245 | 0.451 |
| 1-O-Hexadecyl-2-O-(4Z,7Z,10Z,13Z,16Z,19Z-docosahexaenoyl)-sn-glyceryl-3-phosphorylcholine | -0.192 | -1.270 | 0.451 |
| Octanoylcarnitine | -0.200 | -1.323 | 0.451 |
| Taurine | 0.197 | 1.302 | 0.539 |
| Myristoyl-L-carnitine | -0.167 | -1.097 | 0.544 |
| Hexanoyl-L-carnitine | 0.134 | 0.878 | 0.567 |
| 3-Benzylhexahydropyrrolo[1,2-a]pyrazine-1,4-dione | 0.134 | 0.876 | 0.567 |
| 1-Palmitoylglycerol | 0.131 | 0.854 | 0.567 |
| Erucamide | 0.124 | 0.812 | 0.567 |
| 1-Myristoyl-sn-glycero-3-phosphocholine | -0.128 | -0.837 | 0.567 |
| Lauroyl-L-carnitine | -0.136 | -0.891 | 0.567 |
| Methyl 1H-indol-3-ylacetate | -0.147 | -0.966 | 0.567 |
| 1-Hexadecyl-sn-glycero-3-phosphocholine | -0.153 | -1.001 | 0.567 |
| 1-Palmitoyl-sn-glycero-3-phosphocholine | -0.157 | -1.032 | 0.567 |
| 2,3-Dihydroxypropyl octadecanoate | 0.108 | 0.704 | 0.629 |
| Acetyl-L-carnitine | -0.101 | -0.655 | 0.629 |
| 1-(1Z-Octadecenyl)-2-(5Z,8Z,11Z,14Z-eicosatetraenoyl)-sn-glycero-3-phosphocholine | -0.102 | -0.666 | 0.629 |
| L-Citrulline | 0.173 | 1.141 | 0.639 |
| Palmitoyl sphingomyelin | -0.083 | -0.539 | 0.680 |
| N-(Octadecanoyl)sphing-4-enine-1-phosphocholine | -0.086 | -0.559 | 0.680 |
| Quinolin-2-ol | 0.066 | 0.427 | 0.748 |
| 8-Azabicyclo[3.2.1]octan-3-ol | 0.062 | 0.401 | 0.748 |
| 2-Oxopentanedioic acid | -0.149 | -0.977 | 0.752 |
| Caffeoyl alcohol | -0.137 | -0.896 | 0.780 |
| Urea | -0.048 | -0.313 | 0.797 |
| L-Carnitine | -0.030 | -0.194 | 0.869 |
| Cyclo(leucylprolyl) | 0.025 | 0.161 | 0.873 |
| L-Arginine | 0.097 | 0.630 | 0.877 |
| L-Aspartic acid | -0.092 | -0.600 | 0.877 |
| L-Threonine | -0.103 | -0.672 | 0.877 |
| Citric acid | -0.104 | -0.678 | 0.877 |
| L-Serine | 0.080 | 0.518 | 0.911 |
| Succinic acid | 0.069 | 0.450 | 0.925 |
| D-Aspartic acid | -0.063 | -0.408 | 0.925 |
| Creatine | 0.013 | 0.082 | 0.968 |
| Guanosine | 0.006 | 0.041 | 0.968 |
| Isocitric acid | -0.017 | -0.109 | 0.968 |
| 3-Oxocyclobutanecarboxylic acid | -0.019 | -0.123 | 0.968 |
| Fumaric acid | -0.023 | -0.149 | 0.968 |
| Trans-Aconitic acid | -0.036 | -0.231 | 0.968 |
| D-Fructose | -0.038 | -0.249 | 0.968 |
