# Supplementary material for: Untargeted metabolomics reveals changes in boar sperm and seminal plasma metabolites associated with sexual maturity
Source: J Anim Sci Biotechnol. 2025 Sep 3;16:123. doi: 10.1186/s40104-025-01258-x (PMC12406428; doi:10.1186/s40104-025-01258-x)
Supplement: Supplementary file 6 — Additional file 6: Table S6: Mean decrease accuracy values of annotated metabolites in boar spermatozoa. Note: It is generated from a random forest analysis. [file 40104_2025_1258_MOESM6_ESM.docx]

Table S6: Mean decrease accuracy values of annotated metabolites in boar spermatozoa. It is generated from random forest analysis.

| Metabolites | MeanDecreaseAccuracy |
| --- | --- |
| L-Glutamic acid | 0.048 |
| Homoisovanillic acid | 0.025 |
| N-(1,3-Thiazol-2-yl)benzenesulfonamide | 0.019 |
| DL-Indole-3-lactic acid | 0.014 |
| Decanoyl-L-carnitine | 0.013 |
| Glycerophosphocholine | 0.008 |
| L-Arginine | 0.008 |
| Octanoylcarnitine | 0.006 |
| Fumaric acid | 0.006 |
| Isobutyrylphloroglucinol | 0.006 |
| 3-Indoleacetic acid | 0.006 |
| Succinic acid | 0.005 |
| Trans-Aconitic acid | 0.005 |
| 3'-Galactosyllactose | 0.005 |
| Itaconic acid | 0.004 |
| Heptadecasphing-4-enine | 0.004 |
| L-Threonine | 0.003 |
| Oleamide | 0.003 |
| Isocitric acid | 0.003 |
| DL-Phenylalanine | 0.003 |
| Urea | 0.003 |
| 2,3-Dihydroxypropyl octadecanoate | 0.003 |
| Myo-Inositol | 0.003 |
| L-Aspartic acid | 0.003 |
| Citric acid | 0.003 |
| PC(0-16:0/22:6) | 0.003 |
| Palmitoyl sphingomyelin | 0.002 |
| L-Serine | 0.002 |
| Cyclo(leucylprolyl) | 0.002 |
| 1-Palmitoylglycerol | 0.002 |
| 5'-S-Methyl-5'-thioadenosine | 0.002 |
| 7-Hydroxychromanone | 0.001 |
| 1-Myristoyl-sn-glycero-3-phosphocholine | 0.001 |
| D-Fructose | 0.001 |
| D-Aspartic acid | 0.001 |
| Myristoyl-L-carnitine | 0.001 |
| Valproic acid | 0.001 |
| 1-Palmitoyl-sn-glycero-3-phosphocholine | 7×10^-4^ |
| 8-Azabicyclo[3.2.1]octan-3-ol | 6×10^-4^ |
| N-(Octadecanoyl)sphing-4-enine-1-phosphocholine | 6×10^-4^ |
| 3-Benzylhexahydropyrrolo[1,2-a]pyrazine-1,4-dione | 4×10^-4^ |
| 4-O-.beta.-Galactopyranosyl-D-mannopyranose | 4×10^-4^ |
| 1-(1Z-Octadecenyl)-2-(5Z,8Z,11Z,14Z-eicosatetraenoyl)-sn-glycero-3-phosphocholine | -1.39×10^-5^ |
| Methanesulfonic acid | -2×10^-4^ |
| 3-Oxocyclobutanecarboxylic acid | -4×10^-4^ |
| Acetyl-L-carnitine | -3.7×10^-4^ |
| 1-Formylpyrrolidine-2-carboxylic acid | -4.1×10^-4^ |
| Erucamide | -4.8×10^-4^ |
| Hexanoyl-L-carnitine | -5.8×10^-4^ |
| Quinolin-2-ol | -6.1×10^-4^ |
| Oleoyl ethylamide | -6.7×10^-4^ |
| 4-Formyl-2-hydroxybenzoic acid | -6.9×10^-4^ |
| 1-Hexadecyl-sn-glycero-3-phosphocholine | -9.8×10^-4^ |
| Methyl 1H-indol-3-ylacetate | -0.001 |
| 4,4,7a-Trimethyl-3a,5,6,7-tetrahydro-3H-indene-1-carboxylic acid | -0.001 |
| L-Citrulline | -0.001 |
| Guanosine | -0.001 |
| Glyceric acid | -0.001 |
| Caffeoyl alcohol | -0.001 |
| Hypaphorine | -0.002 |
| Creatine | -0.002 |
| Taurine | -0.002 |
| L-Carnitine | -0.002 |
| Lauroyl-L-carnitine | -0.002 |
| 2-Oxopentanedioic acid | -0.002 |
| Isovaleryl-L-carnitine | -0.003 |
