# Supplementary material for: Untargeted metabolomics reveals changes in boar sperm and seminal plasma metabolites associated with sexual maturity
Source: J Anim Sci Biotechnol. 2025 Sep 3;16:123. doi: 10.1186/s40104-025-01258-x (PMC12406428; doi:10.1186/s40104-025-01258-x)
Supplement: Supplementary file 7 — Additional file 7: Table S7: Mean decrease accuracy values of annotated metabolites in boar seminal plasma. Note: It is generated from a random forest analysis. [file 40104_2025_1258_MOESM7_ESM.docx]

Table S7: Mean decrease accuracy values of annotated metabolites in boar seminal plasma. It is generated from random forest analysis.

| Metabolites | MeanDecreaseAccuracy |
| --- | --- |
| Homoisovanillic acid | 0.038 |
| Glyceric acid | 0.028 |
| 1-Formylpyrrolidine-2-carboxylic acid | 0.026 |
| Glycerophosphocholine | 0.019 |
| N-(Octadecanoyl)sphing-4-enine-1-phosphocholine | 0.019 |
| L-Citrulline | 0.012 |
| Myo-Inositol | 0.012 |
| 7-Hydroxychromanone | 0.012 |
| Oleamide | 0.008 |
| Isobutyrylphloroglucinol | 0.006 |
| 5'-S-Methyl-5'-thioadenosine | 0.006 |
| 3'-Galactosyllactose | 0.004 |
| 1-O-Hexadecyl-2-O-(4Z,7Z,10Z,13Z,16Z,19Z-docosahexaenoyl)-sn-glyceryl-3-phosphorylcholine | 0.004 |
| 3-Indoleacetic acid | 0.003 |
| Oleoyl ethylamide | 0.003 |
| Valproic acid | 0.003 |
| Caffeoyl alcohol | 0.003 |
| 4,4,7a-Trimethyl-3a,5,6,7-tetrahydro-3H-indene-1-carboxylic acid | 0.002 |
| 4-O-.beta.-Galactopyranosyl-D-mannopyranose | 0.002 |
| Taurine | 0.001 |
| Urea | 6.12×10^-4^ |
| N-(1,3-Thiazol-2-yl)benzenesulfonamide | 2.11×10^-4^ |
| L-Threonine | 1.97×10^-4^ |
| 1-Hexadecyl-sn-glycero-3-phosphocholine | 1.59×10^-4^ |
| D-Aspartic acid | 1.34×10^-4^ |
| Methanesulfonic acid | 1.33×10^-4^ |
| Octanoylcarnitine | 6.02×10^-5^ |
| Hypaphorine | -6.52×10^-6^ |
| Isocitric acid | -5.17×10^-5^ |
| L-Carnitine | -7.18×10^-5^ |
| DL-Indole-3-lactic acid | -9.55×10^-5^ |
| 1-(1Z-Octadecenyl)-2-(5Z,8Z,11Z,14Z-eicosatetraenoyl)-sn-glycero-3-phosphocholine | -1.7×10^-4^ |
| Heptadecasphing-4-enine | -2.9×10^-4^ |
| Hexanoyl-L-carnitine | -3.8×10^-4^ |
| 1-Palmitoyl-sn-glycero-3-phosphocholine | -3.9×10^-4^ |
| Decanoyl-L-carnitine | -4×10^-4^ |
| Cyclo(leucylprolyl) | -4.7×10^-4^ |
| Succinic acid | -5×10^-4^ |
| L-Aspartic acid | -5.4×10^-4^ |
| 3-Oxocyclobutanecarboxylic acid | -0.001 |
| Erucamide | -0.001 |
| L-Serine | -0.001 |
| Guanosine | -0.001 |
| Acetyl-L-carnitine | -0.001 |
| 1-Palmitoylglycerol | -0.001 |
| Isovaleryl-L-carnitine | -0.001 |
| Lauroyl-L-carnitine | -0.002 |
| Citric acid | -0.002 |
| 2,3-Dihydroxypropyl octadecanoate | -0.002 |
| 2-Oxopentanedioic acid | -0.002 |
| Creatine | -0.002 |
| 1-Myristoyl-sn-glycero-3-phosphocholine | -0.002 |
| Myristoyl-L-carnitine | -0.002 |
| Itaconic acid | -0.002 |
| L-Glutamic acid | -0.002 |
| 8-Azabicyclo[3.2.1]octan-3-ol | -0.002 |
| 3-Benzylhexahydropyrrolo[1,2-a]pyrazine-1,4-dione | -0.002 |
| Palmitoyl sphingomyelin | -0.002 |
| Fumaric acid | -0.002 |
| D-Fructose | -0.003 |
| L-Arginine | -0.003 |
| Quinolin-2-ol | -0.003 |
| DL-Phenylalanine | -0.003 |
| 4-Formyl-2-hydroxybenzoic acid | -0.003 |
| Methyl 1H-indol-3-ylacetate | -0.004 |
| Trans-Aconitic acid | -0.004 |
| Myristoyl-L-carnitine | -0.001 |
| Citric acid | -0.001 |
| Creatine | -0.001 |
| L-Serine | -0.001 |
| Succinic acid | -0.001 |
| 1-Palmitoyl-sn-glycero-3-phosphocholine | -0.001 |
| 2-Oxopentanedioic acid | -0.001 |
| 3-Oxocyclobutanecarboxylic acid | -0.001 |
| N-(1,3-Thiazol-2-yl)benzenesulfonamide | -0.002 |
| Fumaric acid | -0.002 |
| Guanosine | -0.002 |
| L-Arginine | -0.002 |
| D-Fructose | -0.003 |
| Trans-Aconitic acid | -0.003 |
